# Health, Hope, and Harmony: A Systematic Review of the Determinants of Happiness across Cultures and Countries

**DOI:** 10.3390/ijerph20043306

**Published:** 2023-02-13

**Authors:** Sunitha Singh, Sowmya Kshtriya, Reimara Valk

**Affiliations:** 1 Centre for Entrepreneurship and Innovation, American University in Dubai, Dubai 28282, United Arab Emirates; 2 Department of Psychology, Montclair State University, Montclair, NJ 07043, USA; 3 School of Business and Management, American University in Dubai, Dubai 28282, United Arab Emirates

**Keywords:** happiness, health, hope, harmony, environment, public health

## Abstract

The aim of this study was to review the literature on what constitutes happiness across cultures and countries to advance scholarly knowledge on the happiness construct. A systematic review was conducted to examine the determinants of happiness in samples across cultures and countries. Five different databases, including APA PsycNet, EBSCO-Academic, EBSCO-Business, Project MUSE, and Google Scholar, grey literature, and in-text references from relevant review articles were used. A total of 155 articles were included in the review, encompassing studies from over 100 countries and 44 cultures. Myriad determinants of happiness were found that were placed into three broad categories labeled Health, Hope, and Harmony. The predominant happiness determinants were mental, emotional, and physical well-being, a purposeful holistic work–life balance, nurturing social relationships, caring for self and others, and being in harmony with one’s culture, traditions, community, religion, and environment. This study engendered an “Integrated Model of the Determinants of Happiness” to provide a universally applicable conceptualization of the happiness construct. By examining studies on determinants of happiness across the globe in the past 90 years, this review uncovered that happiness constitutes multiple determinants that fall under three major categories: ‘Health’, ‘Hope’, and ‘Harmony’.

## 1. Introduction

“Happiness is the meaning and the purpose of life, the whole aim, and the end of human existence.” The ancient Greek philosopher Aristotle said these words more than 2000 years ago, and they still ring true today. In Aristotle’s Nicomachean Ethics, happiness is described as the human good that we all aim for its sake alone, and Freud emphasized that happiness is something we strive towards, desire to attain, and maintain throughout our lives [1,2,3]. The 1776 U.S. Declaration of Independence states that all men have a right to “the pursuit of happiness,” where the notion of happiness is equated to the attainment of a worthy life [4].

Moreover, the concept of happiness is gaining increasing popularity within and across cultures [5,6,7], so much so that in recent years, there has been a shift in measuring economic production to measuring happiness as an indicator of social development and individual welfare across nations [8].

Since the advent of the field of positive psychology in the late 1990s, scientific investigations have uncovered happiness as an essential psychological ingredient for optimal human functioning that makes life worth living [9]. Happiness is conceptualized as an appraisal of life [8], a state of mind [6], a psychological state [5], and a positive health indicator [8], and is synonymous with subjective well-being [7,9]. All in all, happiness has been defined in various ways.

Happiness as overall satisfaction with life: Happiness has been conceptualized as an evaluation of life [8], as overall satisfaction with everyday life [10], and as the overall quality of one’s life [11].

Happiness resulting from positive experiences and positive outcomes: Traditionally, happiness has been defined by the experience of more frequent positive affective states than negative ones [12]. Happiness is more than just a personally important goal or a set of pleasant mood states [13], and is related to, precedes, and causes a variety of favorable life outcomes [14]. Furthermore, across studies, happiness has been defined as a positive subjective experience [15].

Happiness as a psychological state of mind, and well-being: Studies have also defined happiness as a psychological state [5], a state of mind [6], a “state of being” [16], a positive attitude toward life [17], a healthy mental status, emotional balance, hope for the future [17], and subjective well-being (SWB) [7], which is the psychological state of well-being, joy, and contentment [14,18]. Happiness as an emotional state is linked to one’s physiological reactions to life events [19] based on the Hedonic Adaptation Theory of Brickman and Campbell [20] and the Set-Point Theory [21].

Happiness as a health indicator: In a study, 785 participants were asked to list associations that came to mind on hearing the word happiness [22]. The participants associated happiness mostly with health and relationships.

Happiness as a transient state: Happiness is defined as a transient mood state of enthusiasm and joy, and it reflects the person’s effect on one’s current state [15].

Despite the myriad conceptualizations of happiness, there are several questions that remain unanswered. The research questions that guided this inquiry were: *Is happiness a temporary state of mind or emotion? Is happiness something we are born with, attain with time, or both? Or Is happiness a period of long-term life satisfaction and general well-being that we all aspire to have in our lives?*

This systematic review attempts to answer these questions and other novel questions that may emerge during the research. The aim of this systematic review [23] is to advance scholarly knowledge by critically reviewing [24,25] the available literature on what constitutes happiness across cultures and countries. In so doing, the reviewers attempt to present a comprehensive conceptualization of happiness that encompasses its determinants coherently and to arrive at a universally applicable conceptualization of happiness supporting the attainment of the following United Nations Sustainable Developmental Goals (SDGs): Good Health and Well-Being (Goal 3), Decent Work and Economic Growth (Goal 8), and Responsible Consumption and Production (Goal 12).

## 2. Materials and Methods

### 2.1. Literature Search and Study Selection

**Figure 1:**
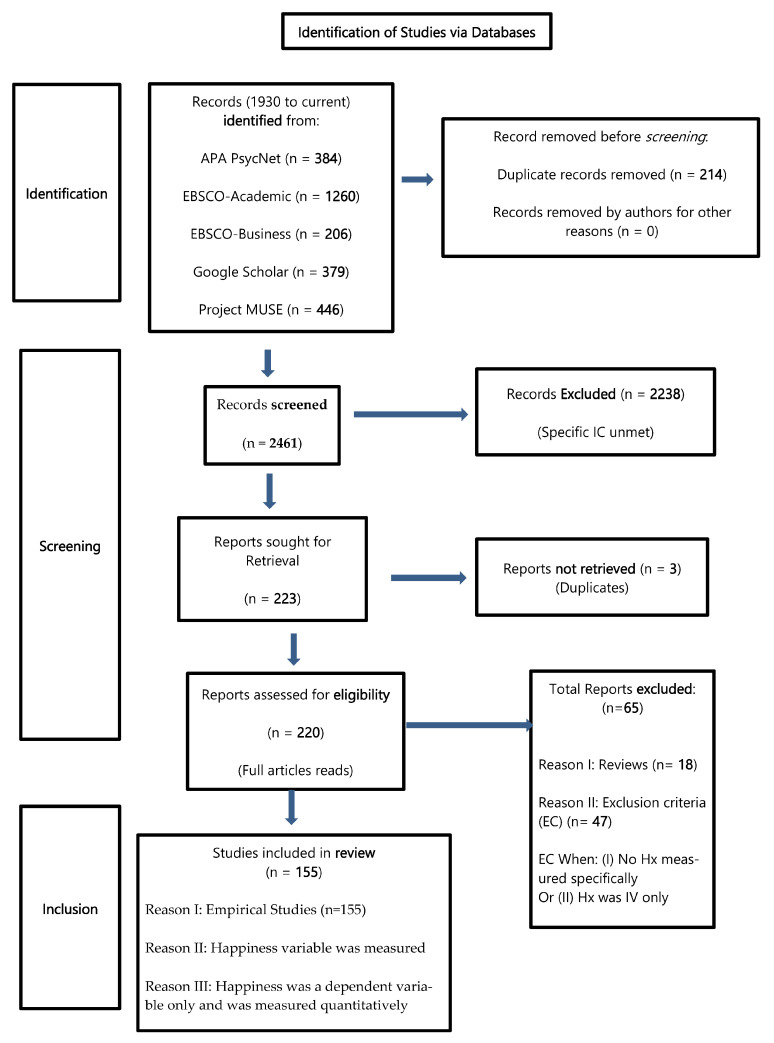
PRISMA flow diagram of the systematic review performed that included searches of databases (19 May to 30 July 2020). Note. Hx: happiness variable, IV: independent variable.

A systematic review [23], was conducted based on the Preferred Reporting Items for Systematic Reviews and Meta-Analyses (PRISMA model, Figure 1) [26], and the Critical Appraisal Skill Program (CASP) Checklist [23,24,25]. These protocols were followed to examine the literature on what constitutes happiness across cultures and countries.

### 2.2. Articles Search and Counts

In a meta-analysis [27] (p. 2695), the following keywords that were derived [28] were highlighted, which included terms such as, “happiness” OR “fulfillment” OR “satisfaction” OR “subjective well-being” OR “meaning” OR “pleasure.” Thus, for this systematic review [23], searches were conducted using the Boolean search strategy that utilized similar keywords, “happiness” OR “well-being” OR “health” OR “life-satisfaction” OR “hope” OR “pleasure” OR “harmony.” The justification for the use of these keywords was to examine happiness and its underlying determinants. Using these keywords, initial studies were identified from the following databases: APA PsycNet—384 studies, EBSCO Academic—1260 studies, EBSCO Business—206, Google Scholar—379, and Project MUSE—446. A total number of 2675 studies relating to happiness and its underlying determinants were shortlisted for review.

The procedure for shortlisting included first reviewing titles that examined happiness, after which, the corresponding abstracts were read. Other published works such as book chapters, case studies, commentaries, or grey literature were initially screened but excluded due to the lack of focus on their empirical quality in assessing the happiness variable. In total, 2238 articles were removed that did not meet the authors’ generic and the three specific inclusion criteria. Furthermore, 223 articles were sought for retrieval using Mendeley research software, at which point, 3 were further removed for overlaps found (Figure 1).

For consistency in the search and quality assurance, all searches were performed using specific inclusion criteria, (i) the happiness variable was measured specifically within the study, (ii) happiness was a dependent variable only and not an independent variable, and (iii) happiness was measured quantitatively. Based on the search results, the inclusion criteria for the literature search and study selection included selecting studies, (i) between 1930 and the present that included the oldest empirical study of happiness [29], (ii) the article type that was peer-reviewed empirical research, and scholarly journals with an impact factor closer to 10 (between 8 and 10), and (iii) all studies had at least one of the search keywords mentioned above. The data were sought to arrive at the underlying determinants of happiness across cultures and countries, through the investigation of the concept of happiness. The extraction criteria were based on the PRISMA model of systematic reviews (Figure 1) [23,26].

Identification: During the identification stage, an initial 2675 peer-reviewed articles related to happiness from 1930 to current (90 years) were found. Before screening, a total of 214 duplicate articles were removed from articles downloaded from four databases (Table 1). In total, 14 duplicate articles were found in APA PsycNet, 43 duplicate articles were found in EBSCO Academic, 21 duplicate articles were found in EBSCO Business, and 136 articles were found in the Project MUSE database (Table 1).

Screening: During the screening stage, a total of 2461 abstracts were selected after removing 214 redundancies (overlaps/duplicates). The abstracts for all 2461 articles were downloaded and further screened for articles that aligned with the authors’ generic and specific inclusion criteria. A total of 220 articles were thus chosen as eligible for a detailed systematic review. These were downloaded onto Mendeley’s research software.

A detailed study of each of the 220 articles led to a further exclusion of 65 articles that comprised 18 review articles and 47 articles that were screened out by the authors’ exclusion criteria where (i) no happiness variable was measured specifically within the study, or, (ii) happiness was an independent variable only and not a dependent variable, or, (iii) happiness was only measured qualitatively.

After the studies were identified, duplicate records were removed and screened, and studies were sought for retrieval and assessed for eligibility (PRISMA model, Figure 1), and chosen for review (Figure 1) [26]. In total, 155 studies were shortlisted for review after applying the exclusion and inclusion criteria and the CASP checklist [24]. Studies were selected for a full review if they helped respond to the following questions: What constitutes happiness? Or what contributes to happiness? Or what does happiness consist of?

### 2.3. Critical Appraisal and Assessment Procedure

The critical appraisal [24,25] further involved specific inclusion and assessment criteria that validated each study regarding its thoroughness and credibility. The studies that we included in the systematic review were mainly empirical studies. The three inclusion criteria applied within the study, (i) the happiness variable was measured specifically within the study, (ii) happiness was a dependent variable only and not an independent variable, and (iii) happiness was measured quantitatively. It involved looking at crucial elements such as (i) the study’s purpose, (ii) objectives, (iii) a methodological design that included *p*-value significance testing (*p* ≤ 0.05), (iv) the use of valid and reliable happiness measures that showed high Cronbach’s alpha coefficient values (0.7 or higher) for the population in each study, (v) sample demographics such as size, age, gender distributions, geographic, or ethnic distributions, and (vi) institutional review board-compliant studies. Ranking the research methodology was also performed based on (i) methodology quality—prevent systematic errors, (ii) precision—random errors (width of confidence around the results), (iii) external validity—the extent to which we applied results to the target population and, (iv) conclusion—expressed on the bases of exploration of ‘what ifs’ and sensitivity analysis. Finally, the following three exclusion criteria were applied for excluding studies beyond the scope of this systematic review, (i) no happiness variable was measured specifically within the study, (ii) happiness was an independent variable only and not a dependent variable, and (iii) happiness was only measured qualitatively.

The Critical Appraisal Skill Program (CASP) checklist [24] for systematic reviews [23], was used to appraise the studies and extract data. Three broad issues were considered when appraising the studies: (i) Are the results of the study valid? (ii) What are the results? (iii) Will the results help locally? Moreover, based on the 10 questions from the CASP Checklist [24], the reviewers evaluated the three broad issues systematically. The 10 questions comprised some screening questions that were answered with either a ‘yes’ or ‘no’. The 10 questions and their responses were,

i. Whether the review addresses a focused question? Yes, the focused question was to examine what constitutes happiness across cultures and countries.
ii. Did the reviewers look for the right type of papers? Yes, studies with an appropriate study design and set inclusion/exclusion criteria were selected.
iii. Whether all relevant studies were included? Yes, the reviewers looked at the reference list and bibliographic databases that were used in addition to the chosen academic databases to saturate the search on relevant articles pertaining to this study.
iv. Did the reviewers assess the quality of the studies included? Yes, the reviewers considered the rigor of the studies as identified in the inclusion and exclusion criteria.
v. Was it reasonable to combine the results of the studies? Yes, when the results from different studies were similar in terms of the determinants of happiness then the results were combined and shown under the positive and negative associations of these determinants with happiness under the Section 3.
vi. What were the overall results of the review? These are shown in each of the tables under findings and correlation analysis.
vii. How precise were the results? The precision was assessed based on how neatly the outcomes matched the study samples with a low rate of errors.
viii. Are the results of the study valid? *p*-value significance testing (*p* = < 0.05) was considered to ensure the validity of the results in each study.
ix. Will the results help locally? Yes, as studies explored happiness in local contexts such as specific countries.
x. Were all important outcomes considered? Yes, the information gathered was large and comprehensive.

The total number of studies was equally divided amongst the authors for individual manuscript review. Using the CASP checklist, each author assessed a set number of studies (e.g., around 50+ studies per author) and created a table to organize the key points from each study, including study journal, happiness measure, sample size/range, and variable findings (Appendix A: Table A1, Table A2 and Table A3). Using this method, all studies were referenced, noting each study’s participant demographics and the results where happiness was the dependent variable, and its determinants were the independent variables. Thus, out of the initial 2675 articles identified from the databases (Table 1), a final count of 155 empirical studies on happiness was shortlisted and included in the review (Figure 1).

The 155 manuscripts that were included in the review were equally and randomly divided amongst the three reviewers. Each reviewer was responsible for reviewing around 50 articles. All three authors reviewed the literature and read the abstracts and articles. Based on the CASP checklist [24] appraisal and the inclusion and exclusion criteria, the three reviewers agreed on which studies to include or exclude. All three reviewers reached a consensus on the criteria for the studies. Subsequently, each reviewer extracted the data, populated tables, compiled the manuscript findings, organized the references, and formatted the tables for their designated set of studies. All three reviewers collectively discussed and put together the thematic and characterization work and developed the universal model of happiness based on the common determinants that emerged during the systematic review.

Whereas the systematic review was not based on any specific theoretical model, the happiness findings uncovered used many theoretical models ranging from the integrative model of sustainable happiness [14], philosophy and psychological theory, where to the average individual, personal happiness is generally held to be the ultimate aim of all human endeavor [30], the ‘theory of Seligman’ [28], which offers three possibilities: happiness can be reached through pleasure, meaning, or engagement [31], the implicit theory framework of happiness to foster a more complete understanding of the processes underlying well-being [32], the theory of personality, where happiness is a personality variable for which a biological basis can be considered [33], and the theory of positive psychology [14].

## 3. Results

**Figure 2:**
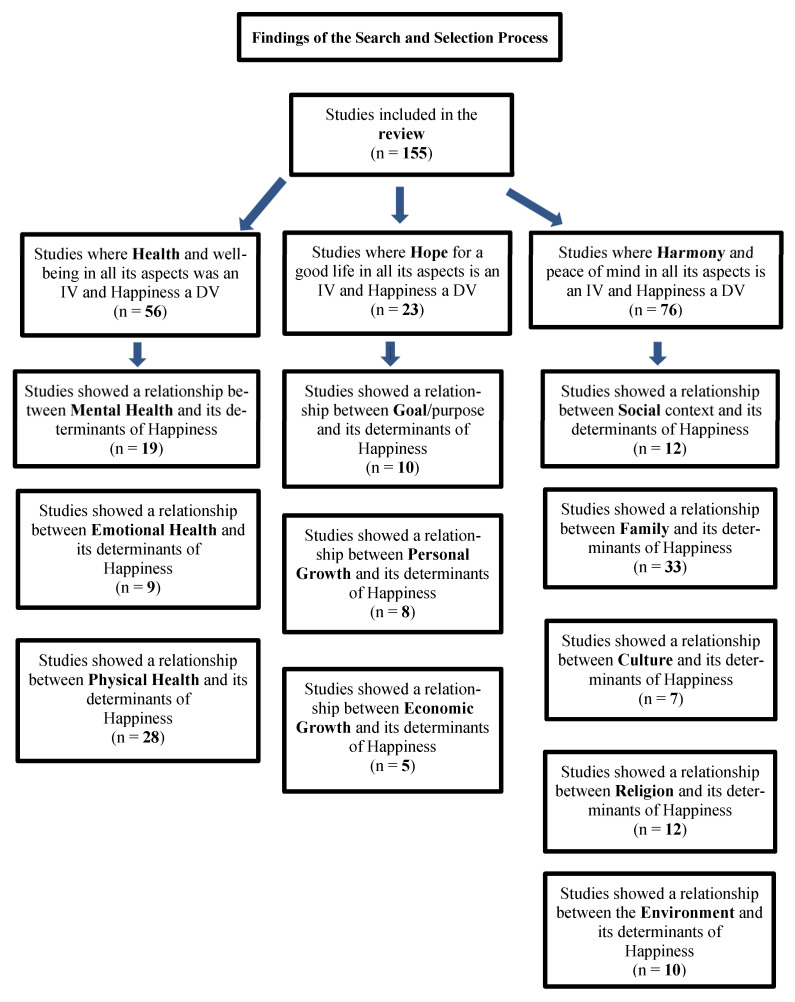
Process flow diagram of the findings of the search and selection process. Note. IV: independent variable; DV: dependent variable.

The final 155 studies revealed manifold determinants of happiness (Table A1, Table A2 and Table A3). Based on the commonalities between these determinants across the findings, we placed these happiness determinants into three broad categories, labeled as, Health, Hope, and Harmony. We conceptualized the first category, “Health”, as a complete state of mental, emotional, and physical well-being. We conceptualized the second category “Hope” as the highest degree of well-founded expectation such as goal achievement and personal and economic growth. Lastly, we conceptualized the third category “Harmony” as a state of being in alignment with aspects of social, familial, cultural, and environmental determinants. The relationships between all categorized determinants and happiness are further elucidated below (Figure 2).

### 3.1. Health and Happiness

A total of 56 studies (Table A1) examined the determinants of health. For precision and simplicity, the Health determinants were categorized under mental, emotional, and physical determinants based on the patterns that emerged across the studies.

#### 3.1.1. Mental Health and Happiness

A total of nineteen studies examined the effects of mental health on happiness. The age range of the participants across these studies was between 10 and 99 years, and the participants were from China, Europe, Germany, India, Iran, Korea, New Zealand, Romania, Spain, Thailand, Turkey, the UK, and the USA. These studies used both female and male participants where a majority were male (55% female, 45% male).

Thirteen studies showed an increase in happiness caused by mental health determinants such as positive general mental health, mindfulness, decreased posttraumatic stress symptoms, creativity, and self-affirmation. Six studies showed a decrease in happiness caused by the determinants of mental health such as adverse mental health outcomes, depression, poor perception of health, lifetime trauma, addiction, and heavy use of screen-based media. These determinants were seen to have a negative relationship with happiness across Asian, American, Black, Hispanic, Native American, Mixed, and White cultures and ethnic groups.

Several studies investigated mental health treatment and its relationship with happiness. A group of researchers showed that meditation (seven-day intensive Vipassana retreat) enhanced happiness [34]. Another research study found that a greater number of sessions per client and decreased post-traumatic stress symptoms were associated with greater counselor happiness [35]. Mindfulness, grit, and coping competence were found to positively predict happiness [9,36,37,38]. Hope and mindfulness were found to share a positive relationship with happiness, and the recognition of new possibilities and personal strength predicted happiness [39,40]. Another group of researchers showed that engaging in spontaneous self-affirmation was related to greater happiness and that self-esteem is an antecedent of happiness [41,42].

Several studies examined negative determinants of happiness. A research study found that a poorer perception of mental health was associated with less happiness [43]. Another study showed daily subtle negative experiences were related to adverse mental health outcomes, such as depression, suicidality, and decreased happiness [44]. A study that examined alexithymia, depression, anxiety, stress, and the relationship of fatigue with happiness, found that decreased posttraumatic stress symptoms were associated with greater counselor happiness [45]. Higher rates of current depression were associated with higher levels of happiness seeking, and greater distress (behavioral health) was associated with lower global happiness [46]. Research showed depression was significantly and negatively associated with pleasure [47], which in turn is associated with happiness. Research revealed an association between creativity and depression and happiness ratings [48]. Other studies examined traumatic life events and happiness. The relationship between lifetime trauma and happiness found that bereavement of a child was associated with lower levels of happiness [49,50]. A negative relationship between stressful life events and happiness was found among humbler people [51].

Studies that examined the association between addiction and happiness found that heavy screen-based media use was associated with less happiness [52], and higher addiction led to lower levels of happiness [53]. A study found internet addiction significantly related to subjective unhappiness [18]. 

#### 3.1.2. Emotional Health and Happiness

A total of nine studies examined the effects of emotional health on happiness. The age range of the participants across these studies was between 9 and 64 years, and the participants were from Asia, Africa, Australia, Canada, China, Europe, India, the Middle East, the UK, and the USA. These studies also used both female and male participants (50% female, 50% male).

All nine studies showed an increase in happiness across countries caused by myriad emotional health determinants that included psychological well-being, Big Five personality traits, humor, gratitude, efficacy, caring climate, and positive emotions. These determinants were seen to have a positive association with happiness.

A study found that psychopathic personality traits such as fearless dominance positively correlated with higher durable happiness and negatively correlated with fluctuating happiness [54]. Fluctuating happiness was described as a sudden increase in happiness, followed by a sudden decrease [54]. Big Five personality traits of extraversion, agreeableness, neuroticism, conscientiousness, and openness to experiences were found to be associated with subjective happiness [33]. Holistic wellness and resilience were found to be determinants of happiness [33,55]. Similarly, psychological well-being was found to have a significant positive association with subjective happiness [56]. Research showed a positive association between positive emotions and greater happiness [57]. Positivity predicted positive emotions with greater happiness [14].

Efficacy and a caring climate were positively associated with happiness (emotional health) [58]. Adaptive humor styles (affiliative humor and self-enhancing humor) significantly predicted subjective happiness, whereas maladaptive humor styles (aggressive humor and self-defeating humor) did not strongly predict subjective happiness [59]. Gratitude practice was found to bolster happiness [59].

Various studies investigated psychological determinants of subjective happiness. Three positive psychology determinants that included gratitude visits, three good things in life, and using signature strengths in a new way, were found to increase happiness [28]. Positive psychological intervention improved happiness of patients undergoing in vitro fertilization as a treatment to become pregnant [60].

#### 3.1.3. Physical Health and Happiness

A total of 28 studies examined the effects of physical health on happiness. The age range of participants across these studies was between 5 and 100 years, and the participants were from 44 countries including Africa, Canada, 15 European countries, the Far East, France, Germany, Georgia, Greece, India, Iran, Italy, Netherlands, Nicaragua, Palestine, Poland, South America, Taiwan, the UK, and the USA. These studies also used most male participants (45% female, 55% male).

A total of 16 studies showed an increase in happiness caused by various physical health determinants that included regular physical activity, general physical health, the health of parents, a healthy diet, health insurance, cochlear implantation surgery, and home dialysis, and nine studies showed a decrease in happiness caused by various determinants of physical health such as poor health, disability, handicap, abuse, advancing age, disfigurement, transition to adulthood, older transgender youth, perceived illness, and health problems.

A study by a group of researchers showed that general health is associated with general happiness [61]. Similarly, health was found to positively associate with happiness [22]. Physical activity was associated with higher levels of happiness [62] and increasing the volume of physical activity was found to be associated with higher levels of happiness [63]. Another study found that individuals who are more physically active are happier [64]. A study also found that regular physical activity was associated with greater happiness [65].

Some studies examined severe disability and illness with happiness. A group of researchers found increased levels of perceived illness to be significantly associated with decreased happiness [66]. More health problems and greater perceived seriousness of the health problems/effects were found to be associated with less happiness [67]. Disability was found to be associated with moderate to large drops in happiness over time [68]. Positive meta-stereotype (positive image) and better perceived general health were associated with higher overall happiness, whereas feelings of loneliness and disability/handicap were associated with lower overall happiness [69]. Suffering from a severe disability was associated with less happiness, and higher BMI was associated with steeper declines in happiness [70]. Conversely, greater happiness was also found among handicapped youth vs. control handicapped youth [16]. People with disfigurements were subjectively judged as being less happy [71].

Several studies investigated medical health policies and the perceptions of health and their relation to happiness. A study showed that national health insurance significantly increased happiness [72]. Cochlear implantation surgery was found to increase happiness in mothers of children with hearing loss [73]. Higher levels of happiness were found among home dialysis patients [66]. Another study reported greater happiness post-renal transplant [74]. Use of a microswitch-based program for Rett syndrome (promotes locomotion fluency) was found to increase happiness [75].

Some studies established relationships between age and happiness in general and based on early trauma and stressful events experienced throughout life. Some studies found no significant difference in the happiness levels between children, adolescents, and adults [10,76]. Another study showed individual happiness determined by age and found a U-shaped relationship between age and happiness [77]. Studies also found a trend in the trajectory of happiness from early adulthood to midlife [8,78]; they showed that older adults who experienced traumatic events during childhood vs. after the transition to adulthood exhibited lower subjective happiness; as age increased, happiness levels decreased. Transition to adulthood exhibited lower subjective happiness and happiness showed a downward trend in the older age groups [79,80]. Research showed a negative association between a past-negative time perspective and happiness with aging [81]. A study found that older transgender youth experienced lower happiness than younger patients [82]. Being younger, widowed, or separated from a spouse and experiencing high levels of stress had significant direct effects on diminishing happiness with low levels of health satisfaction [83]. Another study showed that eudaimonia and hedonic happiness remained relatively stable across the lifespan only in the most affluent nations [84]. This showed the role of determinants in the relationship between age and happiness.

### 3.2. Hope and Happiness

Hope was an emergent happiness theme. A total of 23 studies (Table A2) examined the hope-based determinants of happiness, classified into the categories of purpose and goal achievement, personal growth, and economic growth, based on the patterns that emerged across the studies. Within these categories, goal achievement, task performance, a greater set of goals to pursue, the enjoyment of and success at work, life satisfaction, and positive thinking about the future had a positive association with happiness; socioeconomic status, economic scarcity, and unemployment had a positive association with happiness.

#### 3.2.1. Goal Achievement and Happiness

A total of 10 studies identified a range of work- and study-related determinants that influenced happiness through hope for goal achievement. The age range of participants was between 15 and 94 years, and the participants were from 32 countries including Asia, China, Europe, Germany, Iran, and the USA. Both female and male participants were present in these studies, with a majority being female (55% female, 45% male).

All 10 studies showed an increase in happiness caused by the determinants of goal and purpose that included occupation, task performance, goal focus, a greater set of goals to pursue, education, the enjoyment of and success at work, occupational control, compensation, scholastic achievement, self-employment, job training, and need-supplies across different communities.

Mastery-approach goals were found to facilitate higher levels of happiness with task performance than performance-approach goals in conditions of unfavorable social comparisons [85]. Greater elective selection (choosing a particular goal or set of goals to pursue), loss-based selection (selecting goals in the face of resource loss), optimization (enhancing or acquiring resources to achieve a goal), and compensation (reallocating resources towards another goal to maintain functioning at a specific level) were found related to greater happiness [86]. A person’s valence success at a task predicted greater happiness when they succeeded, but greater unhappiness when they failed [87]. High core self-evaluation and needs-supplies fit (congruence between employees’ needs and the rewards received for work) significantly predicted greater happiness [88]. Enjoyment of and success in work and serious hard-working living were determinants contributing to happiness [29]. Job training, cognition, health, social network, and extraversion explained a substantial proportion of variance in happiness [89]. Higher occupational status corresponded to greater happiness [90]. Similarly, more education, higher personal income, and greater occupational control were related to increased happiness (in men) [91]. Job satisfaction in self-employed workers vs. organizational workers is related to greater happiness [92]. Nations with better scholastic achievement performances (mathematics, reading, and scientific literacy) displayed higher happiness scores [93].

#### 3.2.2. Personal Growth and Happiness

A total of eight studies examined the effects of personal growth on happiness. The age range of the participants across these studies was between 18 and 91 years, and the participants were from Ghana, Slovenia, Switzerland, and the USA. Both female and male participants were present in these studies, with the majority being female (55% female, 45% male). The determinants of personal growth on happiness that emerged were life satisfaction, positive thinking about oneself, growth mindsets, opportunities for learning, perceived power, personal meaning, and positive engagement.

All studies showed an increase in happiness caused by personal growth determinants such as personal growth, life satisfaction, positive thinking about oneself, growth mindsets, opportunities for learning, perceived power, personal meaning, and positive engagement.

Emotional intelligence, personal growth initiative, and life satisfaction showed an association with happiness [94]. Growth mindsets led to stronger beliefs in the changeable nature of happiness and were found associated with greater well-being and greater relationship satisfaction [32]. Perceived power was positively related to happiness [95]. Other studies examined the association between meaning, positive engagement in happiness showed that meaning and engagement were positively associated with happiness [31]. Orientation to pleasure, meaning, and engagement (dimension-centered approach) was positively associated with happiness [96]. Rumination inducing messages led to less happiness, whereas hope-inducing messages led to greater happiness [97]. Higher personal mastery and positive health behaviors were positively correlated with happiness [98]. Higher resilience was associated with greater joviality and happiness [99].

#### 3.2.3. Economic Growth and Happiness

A total of five studies examined the effects of economic growth on happiness. These studies employed the following happiness measures: The age range of the participants across these studies was between 15 and 91 years, and the participants were from 32 cultures across 6 continents and 100 countries that included Asia, Africa, America, China, Indonesia, National Survey, Pakistan, Philippines, and Thailand. Both female and male participants were present in these studies, the majority being female (52% female, 48% male).

Three studies showed an increase in happiness caused by the determinants of economic growth that included increased economic growth, socio-economic status, and fiscal decentralization across economically diverse communities. Two studies showed a negative impact on happiness caused by the determinants of economic growth that included less economic freedom, economic scarcity, the earnings of others, unemployment, and economic disparity across communities.

The determinants of economic growth on happiness, such as socioeconomic status, and fiscal decentralization increased happiness (Table A2). Less economic freedom, economic scarcity, the earnings of others, unemployment, and economic disparity were determinants of economic growth that harmed happiness, as reported in the three studies referenced below.

Subjective socioeconomic status and coming from a higher-income country positively correlated with happiness [100]. Rapid economic growth and rises in the price of housing led to greater happiness in older people than the youth [101]. Income did not affect the level of happiness of those who lived in either urban or rural areas [102]. Fiscal decentralization (improved capacity of districts to deliver public services) significantly increased citizen happiness [103].

Other studies looked at the impact of economic scarcity on happiness. Individuals with unemployment and low health status reported lower happiness [104]. A study found less economic freedom was negatively associated with happiness [105]. These studies show that social comparison rather than absolute earnings or economic status has a great influence on the assessment of happiness. Collectively, these studies show the impact of one’s economic status on happiness, whereas lower status has a greater negative impact on happiness.

### 3.3. Harmony and Happiness

Harmony emerged as a happiness determinants category, where 76 studies (Table A3) examined the determinants of harmony on happiness. For precision and simplicity, the harmony determinants were categorized under social, family, culture, and environment determinants based on the patterns that emerged across the studies.

#### 3.3.1. Social Harmony and Happiness

A total of 12 studies examined the effects of social relationships on happiness and found a positive relationship between them. The age range of the participants across these studies was between 16 and 79 years, and the participants were from 34 countries including Asia, the Americas, Spain, Canada, Germany, South Africa, Slovak, Uganda, the UK, and the USA. Both female and male participants were present in these studies, the majority being female (54% female, 46% male).

In total, 11 studies showed an increase in happiness caused by the determinants of social context that included prosocial behavior, social relations, life balance, leisure, social support, sense of community, socializing, developing positive thinking about social groups, nurturing social relationships, and social context. One study showed no significant relationship between social support and happiness.

Study showed that individual-level happiness was determined by social context, i.e., age, education, employer status, and health [106]. Prosocial actions (acts of kindness towards others) led to greater increase in happiness than self-focused actions and neutral behaviors [107]. Prosocial spending was consistently associated with greater happiness [108]. Influence, social relations, life balance, optimism, work, and leisure were all positively associated with happiness levels [15]. Social relations, higher social support, and a sense of community, even online (Facebook), contributed to decreased loneliness and increased happiness [109,110,111]. School belonging mediated the association between social and academic competence and students’ concurrent happiness [112]. However, a study found no significant relationship between social support and happiness [113].

These studies collectively show that a sense of belonging, good social relations, and support are important determinants of happiness. Other studies examined the effect of social activities on happiness. Training programs in happiness that centered on fundamentals such as keeping busy, spending more time socializing, developing positive thinking, and working on a healthy personality demonstrated significant happiness increase over a control group receiving summary instruction in the program [30]. Activities endorsed by happiness seekers included nurturing social relationships, practicing acts of kindness, pursuing goals, practicing religion and/or spirituality, using strategies to cope with stress or adversity, avoiding overthinking and social comparison, practicing meditation, goal evaluation and tracking, savoring the moment, gratitude journaling, thinking optimistically, remembering happy days, and strengthening social relationships [13].

#### 3.3.2. Family Harmony and Happiness

A total of 33 studies examined the effects of harmony in the family on happiness and found a positive relationship between them. The age range of participants across these studies was between 3 and 96 years, and the participants were from Africa, China, Egypt, Europe, the Far East, Iceland, India, Iran, Japan, Korea, New Zealand, Nicaragua, Pakistan, Portugal, South Africa, South America, Spain, the UK, and the USA. These studies used both female and male participants, where the majority were female (65% female, 35% male).

A total of 29 studies showed an increase in happiness caused by the determinants of family, which included family support, family communication, good connections with family, emotional support, home-dwelling elders, time spent with parents, positive mothering, positive marital relationship, entering cohabitation, perceived help from spouse, gender levels, women’s self-esteem, quality of experience in wife’s role, pregnant women, work-family conciliation, higher resilience, and women with higher affective intensity.

Studies that revolved around family communication, emotional support, and family social support, showed that improved subjective happiness led to family happiness [114,115,116,117]. Happiness was found to be positively associated with good connections with family and friends, school, regular exercise, and meals with family [118]. Family social support, i.e., cohesion, expressiveness, and conflict, showed a positive association with happiness [119]. Family communication, family well-being, and gratitude intervention improved family happiness [114,120]. Similarly, family communication, emotional support, and family social support were found leading to improved subjective happiness and family happiness [115].

Some studies examined the relationship between elders and happiness. Highly successful and home-dwelling elders demonstrated significantly higher happiness [121]. Emotional support from parents together with time spent with parents had the largest positive influence on happiness [122]. Higher perceptions of work–family conciliation predicted higher happiness [123]. Positive mothering led to increased joy and pleasure [124] and emotional deregulation [114].

Several studies examined marital relationships and happiness. Studies found that success in dealing with marriage contributed to happiness [29]; and that a positive relationship existed between marriage and happiness [125]. Higher happiness was found in a balanced marriage [126]; while fewer difficulties in a marital relationship status positively related to happiness and showed that perceived help from the spouse increased partners’ happiness [127]. A more balanced marriage with intra-couple education (both husband and wife are well-educated) demonstrated higher happiness [126]. Unhappily married couples showed a deficit in problem-solving, in more unresolved problems, less involvement with one another, and less shared sexuality [128]. Less happiness in marriage was caused by viewing explicit sexual movies [129]. Other forms of close, intimate relationships also contributed to happiness. Studies found that entering cohabitation is as beneficial as entering marriage and contributed to a peoples’ happiness [19,130].

Moreover, successful marital and parental relations were also positively associated with happiness. Therapist contact programs improved marital happiness [131]. In a study that was conducted in the USA with a racial/ethnic composition of the total enrolled sample that included both mothers and children, with 18% African American/Black, 79% Latino/Hispanic, and 1% of mixed racial/ethnic background, found that children of mothers living with HIV who underwent the Teaching, Raising, and Communicating with Kids (TRACK) program, exhibited increase in happiness [132].

Some studies examined women’s gender role’s impact on happiness. Self-esteem, the number of roles a woman occupied (e.g., paid worker, wife, mother), family income, being a paid worker, and quality of experience in a wife’s role were significantly, positively associated with pleasure [47,133]. Women declared a lower level of happiness compared to men in post-socialist countries [134]. By contrast, women with higher affective intensity than men were as happy as men [79]. Women disagreeing with subservient gender attitudes reported higher happiness [8,135], and research showed males had higher happiness levels than females [136]. Most pregnant women, of maternal age (21–40), and with no smoking history independently correlated with higher happiness [17]. Women with more planned pregnancies, and who had more difficulty in deciding to terminate, experienced lower levels of happiness when it came to deciding about abortions [137]. Most of the women in poverty/victims of intimate partner violence (IPV) showed an optimistic outlook, and higher feelings of social support led to greater happiness [138].

Finally, studies showed a decrease in happiness caused by various determinants of the family such as unhappy couples, negative marital relationships, viewing explicit sexual movies, and women terminating pregnancies [139,140].

#### 3.3.3. Cultural Harmony and Happiness

A total of seven studies examined the effects of culture on happiness. The age range of participants across these studies was between 11 and 90 years, and the participants were from Australia, Canada, China, Germany, Ghana/Sub-Saharan Africa, India, Japan, Malaysia, Netherlands, Rwanda, Taiwan, Thailand, Turkey, and the USA. Both female and male participants were used in these studies, where the majority were female (56% female, 44% male). Six studies showed an increase in happiness caused by the determinants of culture, ethnicity, indigenous culture, multiculturalism, segregation, self-identification, and ethnic identification. Only one study showed a decrease in happiness caused by the determinants of culture.

All studies showed an increase in happiness caused by the determinants of culture that included culture, ethnicity, religion, spirituality indigenous culture, multiculturalism, segregation, self-identification, ethnic identification, faith, forgiveness, religious attendance, tolerance, and spirituality.

Studies found that the characterization of a happy person differed at a cultural level, [12,141], and found culture and polymorphism interacted to influence the perception of happiness. Some studies examined the role of indigenous culture on happiness. Indigenous Australians in remote areas reported higher levels of happiness [142]. Mountain indigenous peoples, females, the elderly, and those who were healthier, wealthier, highly educated, with western beliefs, who received medical benefits, and were without housing problems or financial difficulties were more likely to be happy [143]. Other researchers reported higher levels of happiness among indigenous people [142,143]. Other studies examined the role of identity, multiculturism, and segregation on happiness. National identification, ethnic identification, self-identification, strict identity duality, perceived acceptance, and feeling at home were significantly positively associated with happiness [144]. A positive relationship was found between perceived school multiculturalism and subjective happiness [145]. Decreased segregation was associated with a reduction in happiness among Black populations [146].

#### 3.3.4. Religious Harmony and Happiness

A total of 12 studies examined the effects of religion on happiness. The age range of the participants across these studies was between 11 and 90 years, and the participants were from Australia, Canada, China, Germany, Ghana/Sub-Saharan Africa, India, Japan, Malaysia, Netherlands, Rwanda, Taiwan, Thailand, Turkey, and the USA. Both female and male participants were used in these studies, where the majority were female (56% female, 44% male).

A total of 10 studies showed an increase in happiness caused by the determinants of religion, faith, forgiveness, religious attendance, tolerance, and spirituality. Two studies showed a decrease in happiness caused by spiritual struggles.

Some studies examined the role of religious faith and forgiveness on happiness. The relationship between lifetime trauma and happiness was fully moderated for people who experienced a religious transformation [147]. A significant positive contribution of forgiveness (self, others, situation) was found to lead towards greater happiness [148]. Personal happiness was predicted by active religious involvement and regular attendance to religious services [149,150]. Religious attendance and religiosity were significant positive predictors of happiness [151]. Synagogue attendance, prayer and religious attendance were associated with greater happiness [152]. Happiness positively correlated with the characteristics of tolerance, helpfulness, beliefs, spirituality, responsibility, purposefulness, worthiness, trust, and reliability [153]. Religiousness positively affected with happiness [154]. Practicing Islamic-based gratitude exercises (associating blessings with Allah) raised participants’ happiness levels [155]. Subjective happiness was positively correlated with non-organized religious activity and intrinsic religiosity [156]. Other studies examined the role of spiritual struggles and forgiveness on happiness. More spiritual struggles were associated with less happiness [157]. Specifically, all five types of the religious and spiritual struggles assessed (divine, demonic, interpersonal, moral, and ultimate meaning) correlated significantly negatively with happiness [158].

#### 3.3.5. Environmental Harmony and Happiness

A total of 10 studies examined the effects of the environment on happiness. The age range of participants across these studies was between 18 and 93 years, and the participants were from Australia, Taiwan, the UK, and the USA. Both female and male participants were used in these studies, where the majority were female (60% female, 40% male).

Nine studies showed an increase in happiness caused by the determinants of environment such as ecology, aesthetic neighborhoods, park visitation, green environment, green space, more water, better air quality, quiet neighborhoods, dog ownership, horticulture therapy, and increased environmentally friendly fruit and vegetable consumption. One study showed a decrease in happiness caused by the determinant of environment that included disaster, whereas one study showed a decrease in happiness.

Living in urban vs. rural areas was associated with greater happiness [65]. Park visitation and greater diversity of park activities were found to stimulate happiness [159,160]. Neighborhoods with higher levels of aesthetics, more water, green space, and higher perceived safety were associated with greater happiness [11]. Better air quality/less pollution and quietness in the neighborhood, a higher level of ecological diversity derived from a green environment, diversity of species, and perceived naturalness enhanced happiness [161]. Horticulture therapy that included plant cultivation and plant-related material application significantly improved happiness [162]. Whereas these studies show the relationship between harmony with the local environment and happiness, other studies show the relationship between the foreign environment and happiness. A study showed that travel created short-term happiness through emotional and relational experiences [163]. Harmony with the environment also pertained to environmentally friendly food consumption. Increased fruit and vegetable consumption was predictive of increased happiness [100,164]. Dog ownership increased happiness [165]. This study indicates harmony with species in the immediate environment, important to happiness.

On the negative side, a study showed that environmental disasters significantly decreased happiness [115]. Age, leisure activity engagement, and the earnings of others in the neighborhood were negatively associated with happiness [166,167].

The findings of this systematic review and the subsequent categorization of the predominant emerging happiness determinants resolve previous disputes or indecisive issues about happiness by presenting determinants of happiness that were supported by most studies. Presenting these determinants under three consistent categories of Health, Hope, and Harmony have been depicted in Figure 2 as a process flow diagram.

## 4. Discussion

The purpose of this systematic review was to advance scholarly knowledge by critically reviewing [23,24,25] the literature on what constitutes happiness and the determinants of happiness across cultures and countries. We identified and analyzed 155 empirical studies that examined the effects of different determinants on happiness from over 100 countries and 44 cultures. Based on the patterns that emerged among these studies, the myriad happiness determinants were placed into three major categories: Health, Hope, and Harmony (Appendix A: Table A1, Table A2 and Table A3).

The research questions that guided this inquiry were: *Is happiness a temporary state of mind or emotion? Is happiness something we are born with, attain with time, or both? Or Is happiness a period of long-term life satisfaction and general well-being that we all aspire to have in our lives?* The findings of this study revealed that happiness can be attained, decreased, and increased over one’s lifetime. Happiness can also be a state infused by a period of long-term life satisfaction and general well-being. The happiness determinants derived from the reviewed studies support the transient nature of happiness and its influence by internal and external determinants and circumstances.

Health (mental, emotional, and physical health) and happiness studies show that by focusing on positive health determinants, one may promote the individual’s and society’s well-being for all ages, thus contributing to SDG Good Health and Well-Being (Goal 3).

Hope (goal achievement, personal, and emotional growth) and happiness studies show that by focusing on positive hope determinants, one may promote inclusivity and lifelong learning, sustainable economic growth, and employment opportunity for all ages, supporting SDG Decent Work and Economic Growth (Goal 8).

Harmony (social, familial, cultural, and environmental) and happiness studies show that by focusing on positive Harmony determinants, cities, and human settlements can become inclusive, safe, resilient, and sustainable through sustainable consumption and production patterns, supporting SDG Responsible Consumption and Production (Goal 12) through evidence-based research.

Happiness is conceptualized as an experience that occurs intermittently over one’s lifetime. This fluctuating experience occurs because of the permutations and combinations of various mental, emotional, physical, goal achievement, personal, economic growth, social, family, cultural, religious, and environmental determinants, which can be categorized comprehensively as one’s holistic aspirations for Health, Hope, and/or Harmony. These determinants can positively or negatively impact the experience of happiness and make it fluctuate (either increase or decrease) at different times in one’s life journey. This fluctuating experience of happiness is a result of either a positive or a negative influence of the determinant/s during a given period. For example and to name a few, determinants such as mental depression, addiction, physical disability, economic poverty, social loneliness, separation from a spouse, the death of a loved one, and pollution that have a negative impact vs. determinants such as creativity, humor, relaxation, success at work, doing well at school, financial independence, national pride, religious faith, and pet ownership that have a positive impact can either result in a decrease or increase in one’s happiness experience at a particular given time when the determinant exists. 

**Figure 3:**
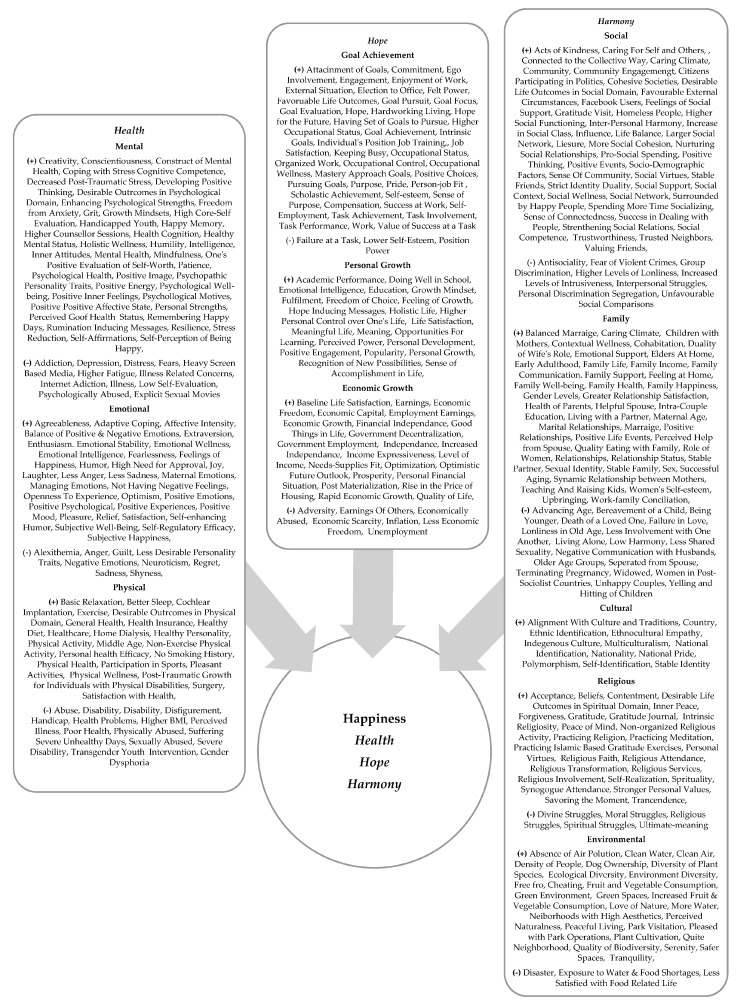
Integrated Model of the Determinants of Happiness. Note. Figure 3 depicts the three key categories of happiness and their underlying determinants. Health: a complete state of mental, emotional, and physical well-being. Hope: the highest degree of a well-founded expectation of goal achievement, personal, and economic growth. Harmony: a state of being in alignment with aspects of social, familial, cultural, religion, and environmental. *+* shows a positive impact. − shows a negative impact.

The conceptualization of happiness has been depicted as an “Integrated Model of the Determinants of Happiness” (Figure 3).

### 4.1. Theoretical Contribution

This review paper contributes to the literature in the interdisciplinary area of environmental health sciences and public health, by presenting an “Integrated Model of the Determinants of Happiness” applicable across cultures and countries. The determinants of happiness are depicted by the authors in the inductively derived “Integrated Model of the Determinants of Happiness” (Figure 3). The model depicts that the three main determinants of happiness, i.e., Health, Hope, and Harmony, are interrelated and interact with each other in a symbiotic manner to determine the happiness of individuals and societies. The “Integrated Model of the Determinants of Happiness” provides a holistic framework that empowers individuals and societies to take control of their happiness (Figure 3).

### 4.2. Implications for Policies and Practice

The systematic review [23] has several implications for policy and practice. The “Integrated Model of the Determinants of Happiness” serves as a foundation and tool for building happiness assessments for measuring the categories of Health, Hope, and Harmony. The results of such assessment can help policymakers and public health practitioners design and implement evidence-based happiness policies and clinical practices that will help individuals and families work on creating their happiness and making societies happier overall. The clinical public health implications are to implement policies and practices at a national level that foster the happiness of individuals as this will result in more productive, vital members of society that can meaningfully contribute to the prosperity of a country. Ultimately, this will lead to beneficial individual, familial, organizational, and economic outcomes, because happy people are more productive people.

### 4.3. Social Implications

The current times of geopolitical upheaval and disruption as well as the COVID-19 pandemic have resulted in instability and uncertainty in the external environment [168,169]. Given these exogenous shock events and global disruptions and distress, “the Integrated Model of the Determinants of Happiness” has great significance, relevance, and impact as it depicts the elements of life that are vital for happiness—something we all strive towards and desire to attain and maintain throughout our lives.

The impacts of anthropogenic determinants on the quality of the environment, the interrelationships between environmental health and the quality of life and happiness, as well as the sociocultural, political, and economic related to happiness across the globe, are noteworthy considerations in fostering the happiness of individuals and societies.

### 4.4. Limitations and Future Research

Some overlap was seen among research studies grouped under the three categories of Health, Hope, and Harmony. However, as a strict demarcation was not possible due to the interrelated and symbiotic nature of the three categories of happiness, the Health, Hope, and Harmony findings were grouped based on the dominant and common underlying determinants that emerged under each category. Future research can address this limitation.

Moreover, this research was conducted before and during the COVID-19 pandemic, whereas happiness studies conducted before the pandemic were analyzed. Therefore, we cannot make any inferences about how happiness has changed among individuals and societies during the pandemic, nor can we make any inferences about whether certain determinants of happiness become more prominent than others during times of crises and adversity.

There is scope for research in this field as each of the categories of happiness can be an area of further research considering external, global exogenous events such as the COVID-19 pandemic. Advanced statistical tools such as confirmatory factor analyses or path modeling can make for a more robust understanding of the pathways to explain happiness, thereby allowing for the statistical generalization of authors’ findings to populations across the globe.

## 5. Conclusions

This systematic review [23] investigated the happiness construct to arrive at a universally applicable conceptualization of the determinants of happiness across the globe. By examining studies on determinants of happiness across 44 cultures and 100 countries in the past 90 years, this systematic review uncovered that happiness is constituted by multiple determinants that can be placed into three main categories: ‘Health’, ‘Hope’, and ‘Harmony’. The happiness determinants are one’s mental, emotional, and physical well-being (Health), having a purposeful holistic encompassing work–life balance, nurturing social relationships, caring for self and others (Hope), and being in harmony with one’s culture, traditions, community, and the environment moderated by economic, social, cultural, and environmental conditions that impact individual and societal happiness (Harmony).

## Figures and Tables

**Table 1:** Initial articles were downloaded from five databases.

Databases	Total	Articles Overlap	Total Peer-Reviewed Articles
APA PsycNet	384	14	370
EBSCO–Academic	1260	43	1217
EBSCO–Business Source	206	21	185
Google Scholar	379	0	379
Project MUSE	446	136	310
	2675	214	2461

## Data Availability

The data presented in this study are available in the Appendix A.

## Appendix A

**Table A1:** Health and Happiness, final count (N = 56).

						Health	Hope	Harmony	67 Unique		
Se	Study	Journal	Happiness Measure	Sample	Correlation of Happiness in Findings	Reviewers’ Agglutination of the Findings with the Three Categories Identified			Country	Culture	Quality Score (h5 Median)
**1**	**Abachizadeh, K., Omidnia, S., Hajebi, A., Shekarriz-Foumani, R., and Mohseny, M. (2018). Measuring positive health of Iranians; Finding from Iran social health survey (ISHS).**	**Medical journal of the Islamic Republic of Iran, 32, 1–6.**	**Self-report item using 5-point Likert Scale**	**10,500 participants from Iran (Age Range = 18 and older; 51% Female, 49% Male).**	Males **showed** higher happiness levels **than females. As** age increased, happiness levels decreased. Happiness **was significantly** higher in cities **than in centers of provinces and rural areas**, in never married or married persons **in comparison to those divorced or widowed, in people with** academic education **and** employed persons. The WHO defined different aspects of positive health as life satisfaction, quality of life, happiness, and self-rated health, and developed appropriate scales to measure positive health. Criterion validity of scales with a 40-question national happiness scale was examined in this research. The correlation coefficient of scales with a 40-item happiness scale was between 0.49, 0.53, 0.57, and 0.60 for ‘health status’, ‘happiness’, ‘quality of life’, and ‘life satisfaction’, respectively”.	**+ satisfaction**	**+ quality of life**	**− increased age**	**Iran**	**Iranian**	**44**
**2**	**Abu-Raiya, H., Pargament, K. I., Krause, N., and Ironson, G. (2015). Robust links between religious/spiritual struggles, psychological distress, and well-being in a national sample of American adults.**	**American Journal of Orthopsychiatry, 85(6), 565–575.**	**Subjective Happiness Scale (SHS)**	**2208 participants from the USA (Mean Age = 50.74, Standard Deviation = 19.00, Age Range = 18–96 years; 59% Female, 41% Male).**	**All 5 types of the** religious/spiritual struggles **assessed** (divine, demonic, interpersonal, moral, ultimate meaning) **correlated significantly** negatively **with** happiness.			**− religion, spiritual struggles**	**USA**	**American**	**54**
**3**	**Ansari, H., Ansari Moghaddam, A., Mohammadi, M., and Mahdavifar, N. (2018). Investigating the relationship between happiness and self-confidence with addiction recurrence in addicted people undergoing methadone treatment referred to addiction treatment centers of Zahedan city with an emphasis on the confounders of demographic variables and variables related to drug taking recurrence.**	**World Family Medicine Journal: Incorporating the Middle East Journal of Family Medicine, 99(5832), 1–8.**	**Oxford Happiness Questionnaire (OHQ)**	**250 participants from Iran (Age Range = 25 years below and above; 18% Female; 82% Male).**	Incidence of addiction **was** higher **in people with** lower levels of happiness but was not statistically significant. Correlational analyses revealed that all 5 types of the struggles assessed (i.e., divine, demonic, interpersonal, moral, ultimate meaning) correlated significantly positively with both depressive symptoms and generalized anxiety, and significantly negatively with both satisfaction with life and happiness. **Happiness has a weak correlation with addiction recurrence.**	**− addiction**		**− divine, interpersonal, moral struggles**	Iran	Iranian	**14**
**4**	**Baruch, G. K., and Barnett, R. (1986). Role quality, multiple role involvement, and psychological well-being in midlife women.**	**Journal of personality and social psychology, 51(3), 578–585.**	**Pleasure Scale (PS)**	**238 participants from the USA (Age Range = 35–55 years; 100% Female).**	Self-esteem, number of roles a woman occupied **(e.g., paid worker, wife, mother)**, family income, paid worker, and quality of experience in a wife’s role **were significantly**, positively **associated with** pleasure. Depression **was significantly** negatively **associated with** pleasure. **Happiness or satisfaction are used as indices of well-being**.	**− Depression**	**+ self-esteem,**	**+ family income, roles of women, quality of experience of wife’s role**	USA	**American**	**120**
**5**	**Bieda, A., Hirschfeld, G., Schoenfeld, P., Brailovskaia, J., Zhang, X. C., and Margrave, J. (2017). Universal happiness? Cross-cultural measurement invariance of scales assessing positive mental health.**	**Psychological Assessment, 29(4), 408–421.**	**Subjective Happiness Scale (SHS)**	**20,000** participants from Germany (Mean Age = 26.54, SD = 4.00, Age Range = 18–60 years; 45% Female, 55% Male), Russia (Mean Age = 20.3, SD = 2.4, Age Range = 14–42 years; **35% Female, 65% Male), and China (Mean Age = 19.73, SD = 1.86, Age Range = 14–42 years; 38% Female, 62% Male).**	**On a** cultural level, **the characterization of a** happy person differed. **Traditionally**, happiness is defined by the experience of more frequent positive affective states than negative ones **(Bradburn, 1969)**.	**+ positive affective state, − negative affective state**			Germany, Russia, China	German, Russian, Chinese	**73**
**6**	**Boissoneault, J., Sevel, L., Robinson, M. E., and Staud, R. (2018). Functional brain connectivity of remembered fatigue or happiness in healthy adults: Use of arterial spin labeling.**	**Journal of clinical and experimental neuropsychology, 40(3), 224–233.**	**Visual Analogue Scale (VAS)**	**17 participants from the USA (Mean Age = 22.4, SD = 5.9; 59% Female, 41% Male).**	Greater **functional connectivity between the** inferior frontal gyrus and a cluster including the right fusiform gyrus, right cerebellum, and right lingual gyrus **was associated with** greater happiness ratings. Greater **connectivity between the** cuneus and a cluster including the left occipital pole, the left fusiform gyrus, and the left lingual gyrus **was associated with** lower happiness **ratings. Positive mood induction resulted in mean in-scanner happiness ratings of 46.07 (18.99). H9**	**+ positive mood**			**USA**	**American**	**43**
**7**	**Bojanowska, A., and Zaleski, A. M. (2016). Lay understanding of happiness and the experience of well-being: Are some conceptions of happiness more beneficial than others?**	**Journal of Happiness Studies, 17(2), 793–815.**	**Positive and Negative Affect Schedule (PANAS)**	**785 participants from Poland (Age Range = 13–45 years).**	Health **was** positively associated **with** happiness. In total, **785 participants were asked to list associations that came to mind on hearing the word ‘happiness’. Participants associated happiness mostly with health and relationships.**	**+ health**			Poland	Polish	**82**
**8**	**Bombari, D., Schmid Mast, M., and Bachmann, M. (2017). Felt power explains the link between position power and experienced emotions.**	**Emotion, 17(1), 55–66.**	**Self-report item using 5-point Likert Scale**	**160 participants from Switzerland (Mean Age = 23.52, SD = 3.65; 50% Female, 50% Male).**	Felt power **was** positively related **to** happiness **but** not positional power. **Positive emotions were an aggregate of happiness and interest. Happiness can also be more post-goal oriented (e.g., relieved, relaxed), in which case**, happiness can be labeled as serenity **(Gable and Harmon-Jones, 2008)**.		**+ felt power, − position power**	**+ serenity**	Switzerland	Swiss	**79**
**9**	**Booker, C. L., Skew, A. J., Kelly, Y. J., and Sacker, A. (2015). Media use, sports participation, and well-being in adolescence: Cross-sectional findings from the UK household longitudinal study.**	**American journal of public health, 105(1), 173–179.**	**6 self-report items using overall happiness scale**	**4899 participants from the UK (Age Range = 10–15; 49% Female, 51% Male).**	Heavy screen-based media (SBM) **use associated with** less happiness. Greater participation in sports **was associated with** higher happiness. **The authors determined happiness with life from six questions on how young people felt about schoolwork, their appearance, their family, friends, school, and life as a whole. They observed a linear relationship between greater sports participation and happiness.**	**+ participation in sports, − heavy screen-based media**			UK	British	**126**
**10**	**Cameron, P., Titus, D. G., Kestin, J., and Kestin, M. (1973). The life satisfaction of nonnormal persons.**	**Journal of Consulting and Clinical Psychology, 41(2), 207–214.**	**Self-report questionnaire**	**46 participants from the USA (Age Range = 6–19 years; 50% Females, 50% Males).**	Greater happiness **was found among** handicapped youth **vs. control**. Male handicapped participants **were** more frequently happy **in both the** academic and recess settings. Happiness is a “state of being” concept **(Osorio, 1966).**	**+ handicapped youth**			**USA**	**American**	**83**
**11**	**Catalino, L. I., Algae, S. B., and Fredrickson, B. L. (2014). Prioritizing positivity: An effective approach to pursuing happiness?**	**Emotion, 14(6), 1155–1161.**	**Valuing Happiness Scale (VHS)**	**235 participants from the USA (Age Range = 21–64 years; 76% Female, 24% Male).**	High in prioritizing positivity **predicted positive emotions and** greater happiness. **The integrative model of sustainable happiness (Lyubomirsky, Sheldon, and Schkade, 2005), in which a genetic set point, circumstances, and intentional activities make up a person’s chronic level of happiness, suggesting that engaging in pleasant activities may be the most effective route to increasing happiness.**	**+ positive emotions, pleasant activities**			**USA**	**American**	**79**
**12**	**Chadwick, A. E., Zoccola, P. M., Figueroa, W. S., and Rabideau, E. M. (2016). Communication and stress: Effects of hope evocation and rumination messages on heart rate, anxiety, and emotions after a stressor.**	**Health communication, 31(12), 1447–1459.**	**Self-report item using 5-point Likert Scale**	**127 participants from the USA (Mean Age = 19.50, SD = 1.63, Age Range = 18–27 years; 50% Female, 50% Male)**	Rumination-**inducing messages led to** less happiness. Hope-**inducing messages led to** greater happiness. **Hope evocation messages should increase other positive emotions, such as happiness. The six emotion scales used were hope (hopeful, optimistic, encouraged), fear (fearful, worried, afraid, anxious, scared, distressed), guilt (guilty, ashamed, embarrassed, remorseful), sadness (sad, sorrowful, dreary, blue, down), happiness (happy, glad, pleased, cheerful, joyful), and anger (angry, mad, irritated, annoyed, frustrated).**	**− Rumination-inducing message**	**+ Hope inducing message**		**USA**	**American**	**73**
**13**	**Chatters, L. M. (1988). Subjective well-being evaluations among older Black Americans.**	**Psychology and Aging, 3(2), 184–190.**	**Self-report item using 3-point Likert Scale**	**581 participants from the USA (Mean Age = 19.50, SD = 1.63, Age Range = 55 and older; 63% Female, 37% Male).**	Being younger, widowed, or separated from a spouse, **and** experiencing high levels of stress and **low** levels of health satisfaction **had significant direct effects on** diminishing happiness. **Happiness was directly influenced by stress and reported satisfaction with health**. Health and stress factors are determinants of happiness ratings	**− high stress**		**− being younger, widowed, separated from spouse**	**USA**	**American**	**46**
**14**	**Chaverri, J., Praetorius, R. T., and Ruiz, E. (2018). Counselor happiness: Effects of therapy work with similar trauma.**	**Social Work in Mental Health, 16(4), 419–435.**	**Oxford Happiness Questionnaire (OHQ)**	**153 participants from the USA (Mean Age = 43.35, SD = 12.3, Age Range = 22–69 years; 67% Female, 33% Male).**	Greater numbers of sessions per client **and** decreased posttraumatic stress symptoms **were associated with** greater counselor happiness. **The study found that posttraumatic stress and numbers of client sessions were the most significant predictors of happiness. Happiness has been linked to work performance, in addition to improved relationships, income, and long life (Lyubomirsky, King, and Diener, 2005).**	**+ decreased posttraumatic stress, higher counselor sessions**			**USA**	**American**	**24**
**15**	**Datu, J. A. D., Yuen, M., and Chen, G. (2018). The triarchic model of grit is linked to academic success and well-being among Filipino high school students.**	**School Psychology Quarterly, 33(3), 428–438.**	**Interdependent Happiness Scale (IHS)**	**504 participants from the Philippines (Mean Age = 14.19, SD = 1.55, Age Range = 22–69 years; 56% Female, 44% Male).**	Grit **(perseverance of effort, consistency of interests, and adaptability to situations)** positively **predicted life** interdependent happiness. **Study 2 showed grit positively predicted life satisfaction, positive affect, and interdependent happiness. It also indicates that grit is linked to subjective well-being, interdependent happiness, and optimal psychological health. Interdependent happiness pertains to “global, subjective assessment of whether one is interpersonally harmonized with other people, being quiescent, and being ordinary, and connected to the collective way of well-being” (Hitokoto and Uchida, 2015, p. 214).**	**+ grit, subjective well-being, psychological health**		**+ interpersonal harmony, connected to the collective way**	Philippines	Philippines	**63**
**16**	**Devins, G. M., Mandin, H., Hons, R. B., Burgess, E. D., Klassen, J., Taub, K., … and Buckle, S. (1990). Illness intrusiveness and quality of life in end-stage renal disease: Comparison and stability across treatment modalities.**	**Health Psychology, 9(2), 117–142.**	**Affect Balance Scale (ABS)**	**99 participants from Canada (Mean Age = 41, 42% Female, 58% Male).**	Higher levels of happiness **were found among** home dialysis patients **vs. compared to in-center hemodialysis patients**. Increased levels of perceived illness intrusiveness **(its impact on quality of life) were significantly associated with** decreased happiness. **Quality of life was conceptualized as involving three facets, including satisfaction/happiness, pessimism/illness-related concerns, and depression/distress. Correlations for the satisfaction/happiness indicators showed that increased levels of perceived intrusiveness were significantly associated with decreased levels of life satisfaction, r(95) = −0.25, p < 0.025, positive affect, r(95) = −0.24, *p* < 0.05, and self-esteem, r(95) = −0.37, *p* < 0.01.**	**+ home dialysis, satisfaction, − perceived illness, illness-related concerns, depression, distress**		**− increased levels of intrusiveness**	**Canada**	**Canadian**	**74**
**17**	**Dewar, A. J., and Kavussanu, M. (2012). Achievement goals and emotions in team sport athletes.**	**Sport, Exercise, and Performance Psychology, 1(4), 254–267.**	**The Sport Emotion Questionnaire (SEQ)**	**358 participants from the UK (34% Female, 66% Male).**	Task involvement **was** positively **associated with** happiness. The **results showed that task involvement was related to happiness, pride, and hope positively, and these relationships were mediated by perceived performance. Ego involvement was positively related to happiness (Dewar and Kavussanu (2011)).**		**+ task involvement, pride, hope, ego involvement**		**UK**	**British**	**48**
**18**	**Diener, E., Colvin, C. R., Pavot, W. G., and Allman, A. (1991). The psychic costs of intense positive affect.**	**Journal of personality and social psychology, 61(3), 492–503.**	**Self-report item using 7-point Likert Scale**	**192 participants from the USA (Age Range = 18 and above; 48% Female, 52% Male).**	Persons’ valence success at a task **predicted** greater happiness **when they** succeeded **but** greater unhappiness **when they** failed.		**+ value of success at a task, − failure at a task**		**USA**	**USA**	**120**
**19**	**Durand, G. (2018). Demystification of the relationship between psychopathy and happiness.**	**Journal of Happiness Studies, 19(2), 381–395.**	**Subjective Fluctuating Happiness Scale (SFHS) and Subjective Authentic–Durable Happiness Scale (SA-DHS)**	**597 participants from Europe, the USA, Asia/Middle East, Africa, Canada (Mean Age = 24.19, SD = 5.28; 45% Female, 55% Male).**	**Psychopathic personality traits**—Fearless dominance **was** positively **correlated with** higher durable happiness **and correlated negatively with** fluctuating happiness. Impulsive antisociality **was** negatively **correlated with** durable happiness **and correlated** positively **with** fluctuating happiness. **Research indicates that happiness, or affective well-being, is related primarily to the frequency, not to the intensity, of positive affect (PA). Fearless dominance (PPI-I) was positively correlated with higher durable happiness, the presence of meaning in life, personal growth, and hope, and correlated negatively with fluctuating happiness. Durable happiness includes characteristics such as contentment and inner peace. Fluctuating happiness represents a sudden increase in happiness, followed by a sudden decrease.**	**+ psychopathic personality traits**		**+ contentment, inner peace, − antisociality**	Europe, **USA**, Asia, Middle East, Africa, Canada	European, **American**, Asian, Middle Easter, African, Canadian	**82**
**20**	**Efklides, A., Kalaitzidou, M., and Chankin, G. (2003). Subjective quality of life in old age in Greece: The effect of demographic factors, emotional state, and adaptation to aging.**	**European Psychologist, 8(3), 178–191.**	**Life Satisfaction Index**	**160 participants from Greece (Age Range = 63–100 years; 50% Female, 50% Male).**	More health problems **and** perceived seriousness of the health problems/effects **associated with** less happiness. Happiness is defined as a transient mood state **of enthusiasm and joy that reflects the person’s affect with respect to his/her current state (Campbell et al., 1976).**	**+ joy, enthusiasm, − more health problems**,			Greece	Greece	**55**
**21**	**Fry, M. D., Guivernau, M., Kim, M. S., Newton, M., Gano-Overway, L. A., and Magyar, T. M. (2012). Youth perceptions of a caring climate, emotional regulation, and psychological well-being.**	**Sport, Exercise, and Performance Psychology, 1(1), 44–57.**	**Subjective Happiness Scale (SHS)**	**395 participants from the USA (Mean Age = 11.80, SD = 1.54, Age Range = 9–16 years; 50% Female, 50% Male).**	Negative and positive affective self-regulatory efficacy **and** caring climate **were** positively associated **with** happiness. Hope has been positively associated with academic performance, positive thinking, and adaptive coping methods. **In a similar vein, happiness refers to the extent that individuals perceive that they are in broad general terms a more happy or unhappy person (Snyder et al., 1991).**	**+ self-regulatory efficacy, positive thinking, adaptive coping**	**+ academic performance**	**+ caring climate**	**USA**	**American**	**48**
**22**	**Fujita, F., Diener, E., and Sandvik, E. (1991). Gender differences in negative affect and well-being: the case for emotional intensity.**	**Journal of personality and social psychology, 61(3), 427–434.**	**Self-report item using 6-point Likert Scale**	**136 participants from the USA (Age Range = 18 and above; 48% Female, 52% Male).**	Women **with** higher affective intensity **than men were as** happy as men. **Gender accounted for less than 1% of the variance in happiness. Diener, Sandvik, Pavote, and Gallagher have shown that to the extent that people with a high need for approval report higher levels of happiness.**	**+ affective intensity, high need for approval**			**USA**	**American**	**120**
**23**	**Fullen, M. C., Richardson, V. E., and Granello, D. H. (2018). Comparing successful aging, resilience, and holistic wellness as predictors of the good life.**	**Educational Gerontology, 44(7), 459–468.**	**Self-report item using 3-point Likert Scale**	**200 participants from the USA (Mean Age = 73.72, SD = 9.05, Age Range = 55 and above; 84% Female, 16% Male).**	Holistic wellness **(physical, social, emotional, spiritual, contextual, occupational) and** resilience **predicted** greater happiness. **In this study, 8.5% of the sample met modified criteria for successful aging (SA) and were used as a comparison group with those who did not meet the criteria. Overall, holistic wellness and resilience predicted happiness, life satisfaction, and physical health better than SA alone.**	**+ Holistic wellness, physical wellness, emotional wellness, resilience, physical health**	**+ occupational wellness**	**+ social wellness, contextual wellness, successful aging**	**USA**	**American**	**29**
**24**	**Galambos, N. L., Fang, S., Kahn, H. J., Johnson, M. D., and Lachman, M. E. (2015). Up, not down: The age curve in happiness from early adulthood to midlife in two longitudinal studies.**	**Developmental Psychology, 51(11), 1664–1671.**	**Self-report item using 3-point Likert Scale**	**968 participants from Canada (Age Range = 18 and above; 47% Female, 53% Male).**	**There was an** upward trend **in the trajectory of** happiness **from** early adulthood to midlife. **Happiness, an indicator of subjective well-being, contributes to thriving in work, relationships, and health, as well as longevity (Hoppmann, Gerstorf, Willis, and Schaie, 2011; Lyubomirsky, King, and Diener, 2005). Diener et al. (1999) argued that happiness is an accumulation of positive emotions. Other scholars have characterized happiness as a balance of positive to negative affect (Ryff, 1989), positive affect experienced on a given day (Stone et al., 2010), and happiness with life (Helliwell, Layard, and Sachs, 2013). Authors label the measure (e.g., happiness, positive affect, life satisfaction, well-being).**	**+ health, longevity, positive emotion, balance in emotions, life satisfaction, well-being**	**+ work**	**+ relationships, early adulthood,**	**Canada**	**Canadian**	**87**
**25**	**Gerstorf, D., Hülür, G., Wagner, G. G., Kunzmann, U., and Ram, N. (2018). Terminal change across facets of affective experience and domain satisfaction: Commonalities, differences, and bittersweet emotions at the end of life.**	**Developmental psychology, 54(12), 2382–2402.**	**Self-report item using 7-point Likert Scale**	**864 participants from Germany (Mean Age = 75 years, SD = 12.5; 41% female, 59% Male).**	Suffering **from a more** severe disability **was associated with** less happiness. Higher BMI **was associated with** steeper declines in happiness. Participants living with a partner **were** happier **with family life throughout their last years. Happiness is elicited during goal pursuit and attainment (Lazarus, 1991). Higher perceived personal control over one’s life was associated with more frequent feelings of happiness and more satisfaction with health, leisure, and family life one year prior to death.**	**− suffering, severe disability, higher BMI, satisfaction with health**	**+ goal pursuit, attainment of goals, higher personal control over one’s life,**	**+ living with a partner, leisure, family life**	**Germany**	**German**	**87**
**26**	**Gilbert, P., McEwan, K., Catarino, F., Baiao, R., and Palmeira, L. (2014). Fears of happiness and compassion in relationship with depression, alexithymia, and attachment security in a depressed sample.**	**British Journal of Clinical Psychology, 53(2), 228–244.**	**Fear of Happiness Scale**	**52 participants from the UK (Mean Age = 48.38, SD = 13.75, Age Range = 21–70 years; 69% Female, 31% Male).**	Fears of happiness **were highly** positively **correlated with** alexithymia, depression, anxiety, **and** stress. **Multiple regression analysis revealed that fear of happiness was the best predictor of depression, anxiety, and stress.**	**− fears, alexithymia, depression, anxiety, stress**			**UK**	**British**	**44**
**27**	**Ha, Y. M., and Hwang, W. J. (2014). Gender differences in internet addiction associated with psychological health indicators among adolescents using a national web-based survey.**	**International Journal of Mental Health and Addiction, 12(5), 660–669.**	**Subjective Happiness Scale (SHS)**	**56,086 participants from Korea (Mean Age = 15.2, SD = 0.02, Age Range = 12–18 years; 49% Female, 51% Male).**	Internet addiction **was significantly related to** subjective unhappiness. **Subjective happiness is defined as the** psychological state of well-being, joy, and contentment **(Lyubomirsky 2001). As expected, poor self-rated health, subjective happiness, and depressive symptoms were significantly correlated with Internet addiction in both genders.**	**− internet addiction, psychological well-being, contentment, joy, − poor health**			Korea	Korean	**102**
**28**	**Haczyński, J. (2016). Happy and Healthy Aging. The Analysis of the Relationship Between Age, Health, Education and Happiness on International Social Survey Programme Data.**	**Problemy Zarządzania, 14 (60), 24–39.**	**Self-report item using 4-point Likert Scale**	**45,680 participants across the globe (Europe, the Far East, South America, and Africa), Age Range = 50 and younger, 50 and older.**	**Happiness showed a downward trend in the older age groups. Women declared a lower level of happiness compared to men in post-socialist countries. Positive emotions or subjective happiness could have better impacts on health and quality of life (Frey, 2002). According to that study, happiness depends on three sets of factors:** **• Demographic and personality factors such as age, gender, and family; • Economic factors, in particular unemployment, income, and inflation;** **• Political factors such as the extent of possibilities for citizens to participate in politics, and the degree of governmental decentralization, circumstances, as well as nationality, education, and health.**	**+ positive emotions, subjective happiness, health**	**+ education, − unemployment, inflation, quality of life, government decentralization,**	**+ citizens’ participation in politics, nationality − older age groups women in post-socialist countries**	**Europe**, Far East, South America, **and Africa**	**European**, Far Eastern, South American, **and** African	**15**
**29**	**Hegarty, R. S., Treharne, G. J., Stebbings, S., and Conner, T. S. (2016). Fatigue and mood among people with arthritis: Carry-over across the day.**	**Health Psychology, 35(5), 492–499.**	**Self-report item using 10-point Likert Scale**	**142 participants from New Zealand (Mean Age = 65.60, SD = 10.90, Age Range = 20–84 years; 68% Female, 32% Male).**	Higher fatigue **earlier in the day predicted** lower happiness. The **results showed that happiness increased at mid-morning and then plateaued. The following equation provides an example, where T equals Time, and T + 1 equals the subsequent time point: Happiness T + 1 = P0 + P1(Fatigue) + P2(PainT) + P3(Happiness) + P4 + Frustration T +**	**− higher fatigue,**			New Zealand	New Zealanders	**74**
**30**	**Jamrozik, A., Oraa Ali, M., Sarwer, D. B., and Chatterjee, A. (2019). More than skin deep: Judgments of individuals with facial disfigurement.**	**Psychology of Aesthetics, Creativity, and the Arts, 13(1), 117–129.**	**Self-Assessment Manikin 9-point pictorial scale**	**145 participants from the USA (Mean Age = 35.39; 43% Female, 57% Male).**	People with “disfigurements” **were** subjectively judged as less happy. **The authors suggested that people with “disfigurements” are perceived as having “fewer desirable personality traits” (e.g., emotional stability, conscientiousness), internal attributes (e.g., happiness, intelligence), and social attributes (e.g., trustworthiness, popularity).**	**+ emotional stability, conscientiousness, intelligence; − “disfigurement”, “less desirable”, personality traits**	**+ popularity**	**+ trustworthiness**	**USA**	**American**	**61**
**31**	**Jopp, D., and Rott, C. (2006). Adaptation in very old age: Exploring the role of resources, beliefs, and attitudes for centenarians’ happiness.**	**Psychology and Aging, 21(2), 266–280.**	**Life Satisfaction Index**	**179 participants from Germany (Age Range = 90 and above; 89% Female, 11% Male).**	Job training, cognition, health, social network, and extraversion **explained a substantial proportion of variance in** happiness. **As determinants of happiness, several resources have been investigated. Basic resources (i.e., job training, cognition, health, social network, extraversion) explained a substantial proportion of variance in happiness.**	**+ health, cognition, extraversion**	**+ job training**	**+ social network**	**Germany**	**German**	**46**
**32**	**Krause, N., Pargament, K. I., and Ironson, G. (2017). Does a religious transformation buffer the effects of lifetime trauma on happiness?**	**The International Journal for the Psychology of Religion, 27(2), 104–115.**	**Subjective Happiness Scale (SHS)**	**2851 participants from the USA (Mean Age = 46.5, SD = 17.6, Age Range = 18 and above; 56% Female, 44% Male).**	**The relationship between** lifetime trauma and happiness **was** fully moderated **for people who experienced a** religious transformation. The **data indicated that the magnitude of the relationship between lifetime trauma and happiness is reduced significantly for people who have had a religious transformation but not for those who have not had this type of religious experience. (Charoenwong et al., 2017; Starbuck, 1901)—both studies found positive relationships between religious transformations and happiness, but both studies rely on convenience samples.**	**− lifetime trauma**		**+ religious transformation**	**USA**	**American**	**27**
**33**	**Krause, N., Pargament, K. I., Hill, P. C., and Ironson, G. (2016). Humility, stressful life events, and psychological well-being: Findings from the landmark spirituality and health survey.**	**The Journal of Positive Psychology, 11(5), 499–510.**	**Self-report item using 3 indicators**	**3010 participants from the USA (Mean Age = 46.3, SD = 17.7, Age Range = 18 and older; 56% Female, 44%).**	**The** negative relationship **between** stressful life events **and** happiness **was** reduced **among** people who were humbler. **The data indicate that stress significantly reduces happiness among the study participants with the lowest observed humility score (β = −0.429; *p* < 0.001).**	**+ humility, − stressful life**			**USA**	**American**	**87**
**34**	**Kugbey, N., Atefoe, E. A., Anakwah, N., Nyarko, K., and Atindanbila, S. (2018). Emotional intelligence and personal growth initiative effects on subjective happiness among university students.**	**Journal of Psychology in Africa, 28(4), 261–266.**	**Subjective Happiness Scale (SHS)**	**260 participants from Ghana (Mean Age = 21.72, SD = 3.61; 70% Female, 30% Male).**	Emotional intelligence **and** personal growth initiative **were** positively associated **with** subjective happiness. The **findings suggest that students’ ability to manage and utilize their emotions, as well as their personal growth initiative, contribute significantly to their level of happiness. It is defined by both happiness (which reflects the affective component of well-being) and life satisfaction (which reflects the cognitive component of well-being) (Diener, Suh, and Lucas, 1999; Ngamaba, 2016).**	**+ emotional intelligence, well-being, life satisfaction, managing emotions**	**+ personal growth**		Ghana	Ghanian	**26**
**35**	**Kumar, M. V. (2015). Emotional expressivity, loneliness, and subjective happiness as predictors of psychological wellbeing among the elderly.**	**Indian Journal of Health and Wellbeing, 6(12), 1169–1173.**	**Subjective Happiness Scale (SHS)**	**60 participants from India (Age Range = 60 and above; 50% Female; 50% Male).**	Psychological well-being **was significantly** positively **associated with** subjective happiness. The **results showed a significant positive relationship between subjective happiness and psychological well-being.**	**+ psychological well-being, subjective happiness**			India	Indian	**24**
**36**	**Lathia, N., Sandstrom, G. M., Mascolo, C., and Rentfrow, P. J. (2017). Happier people live more active lives: Using smartphones to link happiness and physical activity.**	***PLoS ONE*** **, *12*(1), e0160589.**	**Self-Report using Affect Grid on Smartphone App**	**12,838 participants from the UK (Age Range = 15–44 years; 43% Female, 54% Male, 3% Unknown).**	**Individuals who are** more physically active **are** happier. **We examined the relationship between physical activity (measured broadly) and happiness using a smartphone application. The findings reveal that individuals who are more physically active are happier. This research suggests that not only exercise but also non-exercise physical activity is related to happiness. Happiness correlated positively with laughing, r(9164) = 0.21, *p* < 0.001, d = 0.43, and negatively with crying, r(9164) = −0.18, *p* < 0.001, d = 0.38**.	**+ physically active, exercise, non-exercise physical activity, laughter**			**UK**	**British**	**278**
**37**	**Lobos, G., Mora, M., del Carmen Lapo, M., Caligari, C., and Schnettler, B. (2015). Happiness and health and food-related variables: Evidence for different age groups in Chile.**	**Suma Psicológica, 22(2), 120–128.**	**Subjective Happiness Scale (SHS)**	**1163 participants from China (Age Range = 20 to 65 years; 65% Female; 35% Male).**	**People with** more unhealthy days, **a** poorer perception of their health, **and less** satisfaction with their food-related lives **were** less happy. **Health-related factors and satisfaction with food-related life (SWFL) are strong predictors of happiness. Happiness in the sense of a personal attribute can serve as a proxy for well-being (Raibley, 2012). We** define happiness as the degree to which someone positively evaluates the overall quality of his or her present, ‘life as a whole’ **(Veenhoven, 1984).**	**− unhealthy days, poorer perception of health**	**+ holistic life**	**− less satisfied with food-related life**	China	Chinese	**32**
**38**	**Lucas, R. E. (2007). Long-term disability is associated with lasting changes in subjective well-being: evidence from two nationally representative longitudinal studies.**	**Journal of personality and social psychology, 92(4), 717–730.**	**Self-report item using 10-point Likert Scale**	**679 participants from Germany (Mean Age = 53.63; 45% Female, 55% Male).**	Disability **was associated with** moderate to large drops in happiness **over time. Findings have led some to suggest that people are able to adapt to almost any life circumstance and that long-term levels of happiness cannot change. Disabilities can have strong effects on people’s happiness.**	**− disability**			**Germany**	**German**	**120**
**39**	**Matsunaga, M., Masuda, T., Ishii, K., Ohtsubo, Y., Noguchi, Y., Ochi, M., and Yamasue, H. (2018). Culture and cannabinoid receptor gene polymorphism interact to influence the perception of happiness.**	**PLoS ONE, 13(12), 1–17**	**Subjective Happiness Scale (SHS)**	**259 participants from Japan (Mean Age: 19.51, Age Range: 18−28 years; 61% Female, 39% Male) and 181 participants from Canada (Mean Age: 19.47, Age Range: 17−28 years; 78% Female, 22% Male).**	Culture **and** CNR1 polymorphism **interacted to influence the** perception of happiness. **The** subjective happiness level **was the** highest **in** Japanese individuals with the CC genotype, **whereas in** Canadian participants, **it was the highest in individuals** with the TT genotype. **Furthermore, the effects of the *CNR1* genotype on situation-specific happiness were also different between the groups.** Happiness accompanied by being surrounded by happy people **was the** highest **among** Japanese individuals with the CC genotype, **whereas among** Canadian individuals, **it was the** highest in TT genotype carriers. Genotypes on happiness might differ among different cultures because the concept of happiness varies by culture. In Japan, favorable external circumstances determine the concept of happiness, and in Canada, the concept of happiness centers on positive feelings.	**+ positive feelings,**		**+ culture, surrounded by happy people, favorable external circumstances**	Japan	Japanese	**278**
**40**	**Mujcic, R., and J. Oswald, A. (2016). Evolution of well-being and happiness after increases in consumption of fruit and vegetables.**	**American Journal of Public Health, 106(8), 1504–1510.**	**Self-report item using 6-point Likert Scale**	**12,385 participants from Australia (Age Range = 15–93).**	Increased fruit and vegetable consumption **was predictive of** increased happiness. **Increased fruit and vegetable consumption was predictive of increased happiness, life satisfaction, and well-being. The regression equations reveal that fruit and vegetable consumption in the current year is predictive of higher well-being—measured either as life satisfaction or as happiness—in the future even after control for current well-being.**	**+ life-satisfaction, well-being**		**+ increased fruit and vegetable consumption**	Australia	Australian	**126**
**41**	**Murphy, D. A., Armistead, L., Marelich, W. D., Payne, D. L., and Herbeck, D. M. (2011). Pilot trial of a disclosure intervention for HIV+ mothers: The TRACK program.**	**Journal of consulting and clinical psychology, 79(2), 203–214.**	**Happiness and Satisfaction Scale**	**80 participants from the USA (Mean Age = 8.7, SD = 2.0, Age Range = 6–12 years; 37% Female, 63% Male).**	Children **of mothers living with HIV who underwent the** Teaching, Raising, Additionally, Communicating with Kids (TRACK) program **exhibited** increases in happiness. **For those in the intervention group, happiness increased and greater freedom from anxiety was noted from baseline to 3 months.**	**+ greater freedom from anxiety**		**+ children with mothers**	**USA**	**American**	**83**
**42**	**Ogle, C. M., Rubin, D. C., and Siegler, I. C. (2013). The impact of the developmental timing of trauma exposure on PTSD symptoms and psychosocial functioning among older adults.**	**Developmental psychology, 49(11), 2191–2209.**	**Subjective Happiness Scale (SHS)**	**1995 participants from the USA (mean Age = 60.83, SD = 1.57; 31% Female, 69% Male).**	Older adults who experienced traumatic events during childhood **vs. after the transition to adulthood exhibited** lower subjective happiness. **Older adults whose most distressing trauma occurred during childhood reported lower subjective happiness (M = 5.11, SD = 1.18) compared to those whose most distressing trauma occurred in young adulthood (M = 5.44, SD = 1.10), midlife (M = 5.48, SD = 1.08), and older adulthood (M = 5.51, SD = 1.00). The mean (SD) for the adolescence exposure group was 5.27 (1.17). Similar results emerged for psychosocial indices associated with successful adaptation following trauma exposure, including subjective happiness, social support, and coping ability.**	**− older adults with traumatic experiences + subjective happiness, coping ability**		**+ social support**	**USA**	**American**	**87**
**43**	**Peltzer, K., and Pengpid, S. (2017). Dietary consumption and happiness and depression among university students: A cross-national survey.**	**Journal of Psychology in Africa, 27(4), 372–377.**	**Four-item Happiness Scale**	**18,522 participants from 28 countries from Asia, Africa, and the Americas (Mean Age = 20.9, SD = 2.4, Age Range = 17–30 years; 58% Female, 42% Male).**	**The amount of** fruit and vegetable consumption **was** positively **associated with** happiness. **The influence of fruit and vegetable consumption, sociodemographic, and health-related factors on happiness and depression scores were analyzed with a series of multiple linear regression models. The results indicate that the amount of fruit and vegetable consumption was positively associated with happiness and inversely associated with depression.**	**+ health-related factors, − depression**		**+ fruit and vegetable consumption, sociodemographic factors**	28 countries from Asia, Africa, and the Americas	29 cultures from Asia, Africa, and the Americas	**26**
**44**	**Pishgar, F., Soleyman-Jahi, S., Pishgar, F., Eftekhar Ardebili, H., Jamal, A., and Arab, A. (2016). Level of happiness and its determining factors in pregnant women: a cross-sectional study.**	**Journal of Reproductive and Infant Psychology, 34(5), 431–441.**	**Oxford Happiness Questionnaire (OHQ)**	**200 participants from Iran (Mean Age = 28.71, SD = 4.73; 100% Female).**	Most pregnant women, maternal age (21–40), **and** no smoking history **independently correlated with** higher happiness. **Happiness provides a positive attitude toward life, a healthy mental status, emotional balance, hope for the future, and better sleep (Sabatini, 2014).**	**+ no-smoking history, healthy mental status, emotional balance, better sleep, positive attitude towards life**	**+ hope for the future**	**+ pregnant women, maternal age**	**Iran**	**Iranian+L35:L36**	**41**
**45**	**Ren, Q., and Ye, M. (2017). Losing children and mental well-being: evidence from China.**	**Applied Economics Letters, 24(12), 868–877.**	**Center for Epidemiological Studies Depression Scale (CES-D Scale)**	**27,992 participants from China (Mean Age = 61.77, SD = 9.7, Age Range = 45 and older; 50% Female, 50% Male).**	The bereavement of a child **was associated with** lower levels of happiness. **The bereavement of a child is associated with lower levels of mental health and happiness and higher levels of loneliness, and these effects are stronger when all their children are lost.**	**− lower levels of mental health**		**− losing/bereavement of a child, higher levels of loneliness**	**China**	**Chinese**	**52**
**46**	**Richards, J., Jiang, X., Kelly, P., Chau, J., Bauman, A., and Ding, D. (2015). Don’t worry, be happy: cross-sectional associations between physical activity and happiness in 15 European countries.**	**BMC public health, 15(1), 1–16**	**Self-report item using 6-point Likert Scale**	**11,637 participants from 15 European countries (Age Range = 15 and older).**	Increasing physical activity volume **was associated with** higher levels of happiness. **Happiness is an example of a positive construct of mental health that may be promoted by physical activity and could increase resilience to emotional perturbations. Logistic regression was used to examine the association between happiness and physical activity volume adjusted for sex, age, country, general health, relationship status, employment, and education. Happiness was also associated with “a lot” of leisure physical activity (OR = 1.15 [1.02–1.30])**	**+ increasing physical activity, positive construct of mental health, resilience, lot of leisure activity, general health**	**+ employment, education**	**+ country, sex, relationship status**	15 European Countries	16 European Cultures	**131**
**42**	**Ogle, C. M., Rubin, D. C., and Siegler, I. C. (2013). The impact of the developmental timing of trauma exposure on PTSD symptoms and psychosocial functioning among older adults.**	**Developmental psychology, 49(11), 2191–2209.**	**Subjective Happiness Scale (SHS)**	**1995 participants from the USA (mean Age = 60.83, SD = 1.57; 31% Female, 69% Male).**	Older adults who experienced a traumatic event during childhood **vs. after the transition to adulthood exhibited** lower subjective happiness. **Older adults whose most distressing trauma occurred during childhood reported lower subjective happiness (M = 5.11, SD = 1.18) compared to those whose most distressing trauma occurred in young adulthood (M = 5.44, SD = 1.10), midlife (M = 5.48, SD = 1.08), and older adulthood (M = 5.51, SD = 1.00). The mean (SD) for the adolescence exposure group was 5.27 (1.17). Similar results emerged for psychosocial indices associated with successful adaptation following trauma exposure, including subjective happiness, social support, and coping ability.**	**− older adults with traumatic experiences + subjective happiness, coping ability**		**+ social support**	**USA**	**American**	**87**
**47**	**Rocca, C. H., Kimport, K., Roberts, S. C., Gould, H., Neuhaus, J., and Foster, D. G. (2015). Decision rightness and emotional responses to abortion in the United States: A longitudinal study.**	**PLoS ONE, 10(7), 1–16**	**Self-report item using 8-point Likert Scale**	**667 participants from the USA (Age Range = 15 and older; 100% Female).**	Women with more planned pregnancies **and who had** more difficulty deciding to terminate **experienced** lower levels of happiness **when it came to deciding about abortions. The participants completed semiannual phone surveys to assess whether they felt that having the abortion was the right decision for them; negative emotions (regret, anger, guilt, sadness) about the abortion; and positive emotions (relief, happiness).**	**− negative emotions, regret, anger, guilt, sadness, + relief**		**− women with planned pregnancies, deciding to terminate pregnancies, abortions**	**USA**	**American**	**278**
**48**	**Saki, N., Yadollahpour, A., Moniri, S., Karimi, M., Bayat, A., Abshirini, H., and Nikakhlagh, S. (2017). Investigating the impacts of cochlear implantation on the happiness and self-esteem of mothers of children with severe hearing loss.**	**International Journal of Mental Health and Addiction, 15(2), 288–294.**	**Oxford Happiness Questionnaire (OHQ)**	**40 participants from Iran (Mean Age = 27, SD = 5.2).**	Cochlear implantation surgery increases happiness **in mothers of children with hearing loss. A significant difference was observed between the happiness and self-esteem scores before and after surgery (*p* < 0.001). Cochlear implantation surgery increases happiness and self-esteem in mothers of children with hearing loss.**	**+ cochlear implantation surgery**	**+ self-esteem**		**Iran**	**Iranian**	**102**
**49**	**Seligman, M. E., Steen, T. A., Park, N., and Peterson, C. (2005). Positive psychology progress: empirical validation of interventions.**	**American psychologist, 60(5), 410–421.**	**Steen Happiness Index (SHI)**	**577 participants from the USA (Age Range = 35 to 54 years; 58% Female, 42% Male).**	Three positive psychology interventions—**gratitude visits, three good things in life, using signature strengths in a new way**—increased happiness. **Fulfilling—contributes to individual fulfillment, satisfaction, and happiness broadly construed better-defined routes to “happiness” (Seligman, 2002): (a) positive emotion and pleasure (the pleasant life); (b) engagement (the engaged life); and (c) meaning (the meaningful life).**	**+ positive psychology interventions, positive emotions, pleasure**	**+ good things in life, engagement, meaningful life**	**+ gratitude visit**	**USA**	**American**	**142**
**50**	**Stasolla, F., Caffò, A. O., Perilli, V., Boccasini, A., Damiani, R., and D’Amico, F. (2018). Fostering locomotion fluency of five adolescents with Rett syndrome through a microswitch-based program: contingency awareness and social rating.**	**Journal of Developmental and Physical Disabilities, 30(2), 239–258.**	**Behavioral Measure-recording indices of happiness, i.e., smiling, laughing, excited, energized body, arm and leg movements with or without vocalizations.**	**5 participants from Italy (Age Range = 13–17 years.**	**Use of a** microswitch-based program for Rett syndrome **(promotes locomotion fluency)** increased happiness. **Researchers usually refer to behavioral signs of happiness (i.e., smiling, laughing, excited body, and arms and leg movements with or without vocalizations) labeling them indices of happiness and considering them as an outcome measure of the participants’ quality of life.**	**+ microswitch-based programs, quality of life**	**+ quality of life**		Italy	Italian	**30**
**51**	**Van Tongeren, D. R., and Burnette, J. L. (2018). Do you believe happiness can change? An investigation of the relationship between happiness mindsets, well-being, and satisfaction.**	**The Journal of Positive Psychology, 13(2), 101–109.**	**Subjective Happiness Scale (SHS)**	**277 participants from the USA (39% Female, 61% Male).**	Encouraging growth mindsets led to stronger beliefs in the changeable nature of happiness. Happiness growth mindsets **were** associated **with** greater well-being **and** greater relationship satisfaction. **In the current work, we extend the** implicit theory framework **to happiness to foster a more complete understanding of the processes underlying well-being appraisals and subsequent satisfaction with relationships, jobs, and life more generally. Happiness is associated with well-being and related satisfaction outcomes.**	**+ growth mindsets, greater well-being**	**+ satisfaction outcomes**	**+ stronger beliefs, greater relationship satisfaction**	**USA**	**American**	**87**
**52**	**Veni, R. K., Gomes, R. F., and Aurora, A. P. (2018). Differences in happiness and emotional intelligence among adolescents and adults.**	**Indian Journal of Health and Wellbeing, 9(1), 115–117.**	**Oxford Happiness Questionnaire (OHQ)**	**150 participants from India (Age Range = 13 and older).**	No significant difference **was found in** the happiness levels between adolescents and adults. Happiness is about our lives as a whole; **it includes the fluctuating feelings we experience every day but also our overall satisfaction with life. It is influenced by our genes, upbringing, and our external situation. However, crucially, it is also heavily influenced by our choices, our inner attitudes, how we approach our relationships, our personal values, and our sense of purpose. Happiness is the sum over a lifetime of all specific feelings (Kahneman, 1999).**	**+ inner attitudes**	**+ the external situation, positive choices, sense of purpose**	**+ upbringing, relationships, personal values**	**India**	**Indian**	
**53**	**Witvliet, C. V., Richie, F. J., Root Luna, L. M., and Van Tongeren, D. R. (2019). Gratitude predicts hope and happiness: A two-study assessment of traits and states.**	**The Journal of Positive Psychology, 14(3), 271–282.**	**Keyes’s Flourishing Scale**	**181 participants from the USA (Mean Age = 20.07, SD = 1.19, Age Range = 17–27 years; 83% Female, 17% Male).**	Grateful remembering practices **bolstered** present happiness. **Happiness is the enjoyment of a present good. In the stepwise hierarchical regression analysis reported, forgivingness (β = 0.24, *p* = 0.004), self-control (β = 0.23, *p* < 0.002), and patience (β = 0.04, *p* = 0.656) together accounted for 15% of happiness scores.**	**+ gratitude practices**		**+ gratitude practices,**	**USA**	**Chinese**	**87**
**54**	**Yue, X. D., Liu, K. W. Y., Jiang, F., and Hiranandani, N. A. (2014). Humor styles, self-esteem, and subjective happiness.**	**Psychological reports, 115(2), 517–525.**	**Subjective Happiness Scale (SHS)**	**227 participants from China (Mean Age = 20.90, SD = 1.7, Age Range = 18–28 years; 59% Female, 41% Male).**	Adaptive humor styles (affiliative humor and self-enhancing humor) **significantly** predicted subjective happiness. Maladaptive humor styles (aggressive humor and self-defeating humor) did not **strongly** predict **subjective** happiness. **Adaptive humor styles (affiliative humor and self-enhancing humor) significantly predicted self-esteem and subjective happiness. Subjective happiness was positively associated with affiliative humor (β = 0.20) and self-enhancing humor (β = 0.39).**	**+ affirmative humor**			**China**	**Chinese**	**54**
**55**	**Zhang, S., Zhang, H., Qiu, Z., and Tang, H. (2017). Effects of a positive psychological intervention on the mental health and happiness of patients undergoing in vitro fertilization.**	**Biomedical Research, 28 (9): 4020–4025.**	**Memorial University of Newfoundland Scale of Happiness (MUNSH)**	**200 participants from China (100% Female).**	**A** positive psychological intervention improved the happiness **of patients undergoing in vitro fertilization (IVF). The total happiness score, positive affect score, and positive experience score were significantly higher. Thus, a positive psychological intervention can improve the mental health and happiness of patients undergoing IVF and improve the clinical pregnancy rate.**	**+ positive psychological interventions, positive experience, mental health**			**China**	**Chinese**	**35**
**56**	**Ziapour, A., Khatony, A., Jafari, F., and Kianipour, N. (2018). Correlation of Personality Traits with Happiness among University Students.**	**Journal of Clinical and Diagnostic Research, 12(4), 26–29.**	**Oxford Happiness Questionnaire (OHQ)**	**400 participants from Iran (Age Range = 18–26 years; 38% Female, 62% Male).**	**There was a significant** positive **relationship between** happiness **and** extraversion, agreeableness, neuroticism, conscientiousness, and openness to experiences. **The results of correlation analyses demonstrated that there was a significant positive relationship between happiness (*p* < 0.001) and each of the personality trait dimensions of extraversion (*p* < 0.001, r = 0.594), agreeableness (*p* < 0.001, r = 0.431), neuroticism (*p* < 0.001, r = 0.368), conscientiousness (*p* < 0.001, r = 0.351), and openness to experiences (*p* < 0.001, r = 0.151). According to the** theory of personality **by Eysenck SB et al., happiness is a personality variable for which a biological basis can be considered** [26].	**+ extraversion, agreeableness, openness to experience, conscientiousness, − neuroticism**			**Iran**	**Iran**	**71**

**Table A2:** Hope and Happiness, final count (N = 23).

						Health	Hope	Harmony	42 Unique		
Se	Study	Journal	Happiness Measure	Sample	Correlation of Happiness in Findings	Reviewers’ Agglutination of the Findings with the Three Categories Identified			Country	Culture	Quality Score (h5 Median)
**1**	**Abu-Raiya, H., Pargament, K. I., Krause, N., and Ironson, G. (2015). Robust links between religious/spiritual struggles, psychological distress, and well-being in a national sample of American adults.**	**American Journal of Orthopsychiatry, 85(6), 565.**	**Self-Report**	**2208 American adults.**	**All five types of the religious/spiritual struggles assessed (i.e., divine, demonic, interpersonal, moral, ultimate meaning) correlated significantly negatively with happiness. Correlational analysis revealed that all the five types of the struggles assessed (*, i.e., divine, demonic, interpersonal, moral, ultimate-meaning) correlated significantly positively with both depressive symptoms and generalized anxiety, and significantly negatively with both satisfaction with life and happiness.**			**− religious. Spiritual struggles, moral struggles, interpersonal struggles, divine struggles, demonic struggles**	**America**	**American**	**54**
**2**	**Adelmann, P. K. (1987). Occupational complexity, control, and personal income: Their relation to psychological well-being in men and women.**	**Journal of Applied Psychology, 72(4), 529.**	**1976 Cross-Sectional National Survey**	**948 American adults (Age Range = 21 and over; 35% female, 65%).**	**More education, higher personal income, and greater occupational control were related to increased happiness (in men), and increasing age was related to lower happiness (in women). Stepwise regression results indicate that occupational characteristics (higher occupational complexity, control, and personal income) explain a small but significant proportion of variance in each measure of psychological well-being controlling for the effects of age and education. Occupational characteristics also explain a significant proportion of variance in self-confidence for both men and women and in happiness for men.**		**+ more education, higher personal income, greater occupational control**	**− increasing age**	**America**	**American**	**118**
**3**	**Aknin, L. B., Barrington-Leigh, C. P., Dunn, E. W., Helliwell, J. F., Burns, J., Biswas-Diener, R., ... and Norton, M. I. (2013). Prosocial spending and well-being: Cross-cultural evidence for a psychological universal.**	**Journal of Personality and Social Psychology, 104(4), 635.**	**Subjective Happiness Scale (SHC)**	**627 students from Canada (n = 140; Mean age = 19.95, SD = 3.91, 54% Females, 46% Male) and Uganda (n = 487; Mean age = 21.71, SD = 2.55; 24% and 72% Females, 76% and 28% Males in two separate universities).**	**Testing for causality showed that prosocial spending was consistently associated with greater happiness. Cross-cultural research has shown that the within-country correlations between how much money individuals make, and their happiness may vary according to a country’s average income (e.g., Deaton, 2008; Diener and Biswas-Diener, 2002). This suggests that the link between how individuals spend that money, and their happiness might also differ between poor and wealthy countries.**		**+ earnings**	**+ pro-social spending**	**Canada**	**Canadian**	**120**
**4**	**Burrow, A. L., and Hill, P. L. (2011). Purpose as a form of identity capital for positive youth adjustment**	**Developmental psychology, 47(4), 1196.**	**Subjective Happiness Scale (SHC)**	**107 Students from the USA (Mean age = 15.74, SD = 1.85; 51% Female, 49% Male).**	**Purpose commitment was positively associated with happiness. In the study (n = 110 + n = 398), purpose commitment was positively associated with hope and happiness among adolescents and emerging adults, and indices of well-being. The results showed that purpose commitment fully mediated the relationship between identity and changes in daily positive and negative affect. Overall, the findings suggest that cultivating a sense of purpose in life contributes to well-being.**		**+ purpose, commitment**	**+ stable identity**	**USA**	**American**	**87**
**5**	**Emanuel, A. S., Howell, J. L., Taber, J. M., Ferrer, R. A., Klein, W. M., and Harris, P. R. (2018). Spontaneous self-affirmation is associated with psychological well-being: Evidence from a US national adult survey sample.**	**Journal of Health Psychology, 23(1), 95–102.**	**Self-report item using 5-point Likert Scale)**	**3185 American participants (Mean age = 47.12, SD = 1.13; 52% Female, 48% Male; 67% White, 33% Black/Hispanic/Others).**	**Engaging in spontaneous self-affirmation was related to greater happiness. Engaging in spontaneous self-affirmation was related to greater happiness, hopefulness, optimism, subjective health, personal health efficacy, and less anger and sadness. People who were more likely to spontaneously self-affirm reported more positive and less negative affect including greater happiness, b = 0.14, 95% confidence interval (CI) = [0.08, 0.22], SE = 0.03, t = 4.28, partial = 0.52, *p* < 0.01.**	**+ optimism, subjective health, personal health efficacy, less anger, less sadness, self-affirmation,**	**+ hopefulness**		**Americas**	**Americas**	**79**
**6**	**Fordyce, M. W. (1983). A program to increase happiness: Further studies.**	**Journal of Counseling Psychology, 30(4), 483.**	**Happiness Measures (HM)**	**112 American students (Mean age = 24.5 years, Range = 16–22 years; 54% Female, 46% Male).**	**A training program in happiness that centered on fundamentals including keeping busy, spending more time socializing, developing positive thinking, and working on a healthy personality demonstrated significant happiness increases in a control group receiving summary instructions in the program. In philosophy, in psychological theory, and for the average individual, personal happiness is generally held to be the aim of all human endeavor. Happiness has come to be defined as something much broader in scope than a temporary mood state. Bradburn (1969) hypothesized happiness to be the resulting balance of all positive and negative emotional experiences over a period. Happiness is an emotional state of well-being, and the feeling goes by many names (contentment, fulfillment, self-satisfaction, joy, peace of mind, etc.).**	**+ developing positive thinking, healthy personality, balance of positive and negative emotions, well-being, contentment, self-satisfaction, joy**	**+ keeping busy, fulfillment**	**+ spending more time socializing, peace of mind**	**America**	**America**	**78**
**7**	**Kamarova, S., Chatzisarantis, N. L., Hagger, M. S., Lintunen, T., Hassandra, M., and Papaioannou, A. (2017). Effects of achievement goals on perceptions of competence in conditions of unfavourable social comparisons: The mastery goal advantage effect.**	**British Journal of Educational Psychology, 87(4), 630–646.**	**Two self-report items on 19-point semantic differential scale.**	**201 students (Mean age = 22.53, SD = 6.51; Range = 17–55; 72% Female, 28% Male).**	**Mastery-approach goals facilitated higher levels of happiness with task performance than performance-approach goals in conditions of unfavorable social comparisons. The interactive effects between performance-approach goals and normative information were statistically significant in the two regression analyses that aimed to predict competence or happiness with task performance.**		**+ mastery approach goals, task performance**	**− unfavorable social comparisons**	**Greece**	**Greek**	**56**
**8**	**Kavčič, T. and Avsec, A. (2014). Happiness and pathways to reach it: Dimension-centered versus person-centered approach.**	**Social Indicators Research, 118(1), 141–156.**	**Orientations to Happiness Questionnaire (OTHQ)**	**1142 Adult participants from Slovenia (Mean age = 38.2 years, SD = 15.90, Range = 18 to 91 years; 67% Female, 33% Male).**	**Orientation to pleasure, meaning, and engagement (dimension-centered approach) was positively associated with happiness. The theory of Seligman (2002) offers three possibilities—happiness can be reached through pleasure, meaning, or engagement.**	**+ pleasure**	**+ engagement, meaning**		**Slovenia**	**Slovenian**	**77**
**9**	**Kim, J., Kim, M., and Park, S. H. (2016). Exploring the relationship among posttraumatic growth, life satisfaction, and happiness among Korean individuals with physical disabilities.**	**Psychological Reports, 119(1), 312–327.**	**Subjective Happiness Scale (SHC)**	**212 participants from Korea (Range = 14 to 62 years; 23% Female, 77% Male).**	**The recognition of new possibilities and personal strength predicted happiness. The results of the regression analysis confirmed the research hypothesis that factors of posttraumatic growth are positively associated with life satisfaction and happiness for individuals with physical disabilities.**	**+ personal strength, posttraumatic growth for individuals with physical disabilities**	**+ recognition of new possibilities**		**Korea**	**Korean**	**54**
**10**	**Kirkcaldy, B., Furnham, A., and Siefen, G. (2004). The relationship between health efficacy, educational attainment, and well-being among 30 nations.**	**European Psychologist, 9(2), 107–119.**	**World Database of Happiness**	**Quarter of a million high school students from 32 countries participated in the survey (Age = 15-year-olds).**	**Nations with better scholastic achievement performances (mathematics, reading, and scientific literacy) displayed higher happiness scores. Happiness was consistently related to the three literacy scores (reading, mathematical, and scientific literacy), the magnitude of the association being highest for reading literacy. Veenhoven (2000) concluded that health care has only a minimal impact on subjectively perceived happiness. Veenhoven (2003) reports a total of 330 empirical studies focusing on happiness catalogued in his World Database of Happiness. The construct of well-being and happiness can be assessed simply by asking people to provide an overall judgment of their feeling of happiness (general appraisal of life) or well-being. Veenhoven’s database of happiness scores for those nations included in the current study reveal that those nations with the highest ratings for subjectively perceived happiness include Iceland, followed by Sweden, Australia, Switzerland, Ireland, and the USA. The nations scoring lowest were Russia, Hungary, Brazil, Portugal, Poland, Mexico, and Greece. Veenhoven (2003) lists research findings on satisfaction with life as a whole. From this collection, we used average happiness in nations (means), and happiness-adjusted life years (a combination of average happiness and life expectancy, analogous to disability-adjusted life years).**	**+ health care, feelings of happiness/well-being**	**+ better scholastic achievement**		**32 countries**	**33 countries**	**55**
**11**	**Moza, D., Maricuțoiu, L., and Gavreliuc, A. (2019). Cross-lagged relationships between self-esteem, self-construal, and happiness in a three-wave longitudinal study.**	**Journal of Individual Differences, 40(3), 177.**	**Subjective Happiness Scale (SHC)**	**101 Romanian undergraduates.**	**Self-esteem is an antecedent of happiness. Cross-cultural research established that an interdependent construal of the self is associated with higher self-esteem, which in turn, is associated with increased happiness. Structural equation modeling analyses verified that self-esteem is an antecedent of both happiness and dimensions of independent self-construal. One’s positive evaluation of self-worth precedes one’s self-perception of being a happy and independent person.**	**+ self-perception of being happy**	**+ self-esteem, one’s positive evaluation of self-worth, independence**		**Romania**	**Romanian**	**31**
**12**	**Nelson, S. K., Layous, K., Cole, S. W., and Lyubomirsky, S. (2016). Do unto others or treat yourself? The effects of prosocial and self-focused behavior on psychological flourishing.**	**Emotion, 16(6), 850.**	**Affect Adjective Scale (AAS)**	**472 Adults from the USA (Mean age = 29.95, SD = 11.47; 60% female, 40% Male).**	**Prosocial actions (acts of kindness towards others) led to greater increases in happiness than self-focused actions and neutral behaviors. Mounting evidence suggests that being kind to others (i.e., engaging in prosocial behavior) consistently leads to increases in happiness (Aknin, Hamlin, and Dunn, 2012; Alden and Trew, 2013; Chancellor, Jacobs Bao, and Lyubomirsky, 2015; Layous, Lee, Choi, and Lyubomirsky, 2013; Lyubomirsky, Sheldon, and Schkade, 2005; Mongrain, Chin, and Shapira, 2011; Nelson et al., 2015; Otake, Shimai, Tanaka-Matsumi, Otsui, and Fredrickson, 2006; Pressman, Kraft, and Cross, 2015; Sheldon, Boehm, and Lyubomir-sky, 2012; Weinstein and Ryan, 2010). Hedonic and eudaimonic well-being represent two different ways of pursuing happiness rather than two different types of happiness (Kashdan, Biswas-Diener, and King, 2008). We use the terms happiness, well-being, and flourishing interchangeably.**	**+ hedonic and eudaimonia well-being**	**+ self-focused actions**	**+ prosocial actions, prosocial behavior**	**USA**	**American**	**79**
**13**	**Parks, A. C., Della Porta, M. D., Pierce, R. S., Zilca, R., and Lyubomirsky, S. (2012). Pursuing happiness in everyday life: The characteristics and behaviors of online happiness seekers.**	**Emotion, 12(6), 1222.**	**Authentic Happiness Inventory (AHI)**	**912 adults (Mean age = 45.51, SD = 12.43; Range = 21–83; 77% Female, 23% Male; 87% Caucasian, 13% Asian/Asian-American/Black/Hispanic/Native American/Mixed or unspecified ethnicity).**	***Characteristics and Behaviors*** **—Higher rates of current depression were associated with higher levels of happiness seeking. Most activities endorsed by happiness seekers were nurturing social relationships, practicing acts of kindness, pursuing goals, practicing religion and/or spirituality, using strategies to cope with stress or adversity, avoiding overthinking and social comparison, practicing meditation, goal evaluation and tracking, and savoring the moment. Happiness is more than just a personally important goal or a set of pleasant mood states; a meta-analysis of 225 cross-sectional, longitudinal, and experimental studies found that happiness is related to, precedes, and causes a variety of favorable life outcomes (Lyubomirsky, King, and Diener, 2005). The present studies suggest that real-world happiness strategies have observable unique characteristics and that it is indeed possible to begin to assess the benefits of such practices in real time using innovative technologies. Gratitude journalling, thinking optimistically, remembering happy days, and strengthening social relationships.**	**+ coping with stress, gratitude journal, thinking optimistically, remembering happy days, − depression**	**+ pursuing goals, goal evaluation, favorable life outcomes, − adversity**	**+ nurturing social relationships, acts of kindness, practicing religion, spirituality, practicing meditation, strengthening social relationships, − social comparisons, savoring the moment,**	**Asian/Asian-American/Black/Hispanic/Native American/Mixed or unspecified ethnicity**	**Asian/Asian-American/Black/Hispanic/Native American/Mixed or unspecified ethnicity**	**79**
**14**	**Rana, S., Hariharan, M., Nandine, D., and Vincent, K. (2014). Forgiveness: A determinant of adolescents’ happiness.**	**Indian Journal of Health and Wellbeing, 5(9), 1119–1123.**	**Oxford Happiness Questionnaire (OHQ)**	**200 Indian adolescents (100 early adolescents; Age Range = 13–15, 47% Female, 53% Male, 100 late Adolescents; Age Range = 18–20; 63% Female, 37% Male).**	**There was a significant positive contribution of forgiveness (self, others, situation) that led to greater happiness. Age was also a significant factor for happiness as older adolescents were higher in forgiveness vs. younger adolescents. The results revealed that there were significant contributions of forgiveness and its domains on the happiness of adolescents. Happiness was a composite of life satisfaction (Diener, Lucas, and Scollon, 2006), coping resources (Lyubomirsky, King, and Diener, 2005), positive emotions (Fredrickson, 1998), desirable life outcomes in physical, psychological, social, and spiritual domains in every phase of the life span.**	**+ age, positive emotions, desirable outcomes in physical domain, desirable outcomes in psychological domain**		**+ forgiveness, desirable life outcomes in social domain, desirable outcomes in spiritual domain**	**India**	**Indian**	
**15**	**Rego, A. (2009). Do the opportunities for learning and personal development lead to happiness? It depends on work-family conciliation.**	**Journal of Occupational Health Psychology, 14(3), 334.**	**Affective Well-Being Scale (AWB)—Portuguese version**	**404 individuals from Portugal (Mean age = 27.9 years, SD = 4.7; 27% Female, 73% Male).**	**Higher perceptions on both variables—opportunities for learning and personal development and work—family conciliation predict higher happiness. Pathways to happiness vary across cultures (Diener, Oishi, and Lucas, 2003; Haller and Halder, 2006). Scholars tend to treat happiness as subjective well-being or psychological well-being.**	**+ subjective well-being, psychological well-being**	**+ opportunities for learning, personal development**	**+ work–family conciliation**	**Portugal**	**Portuguese**	**104**
**16**	**Šeboková, G., Uhláriková, J., and Halamová, M. (2018). Cognitive and social sources of adolescent well-being: Mediating role of school belonging.**	**Studia Psychologica, 60(1), 16–29.**	**EPOCH Measure of Adolescent Well-being**	**139 Slovak students (Mean age = 15.63, SD = 1.15; 67% Female, 33% Male).**	**School belonging mediated the association between social and academic competence and students’ concurrent happiness. The results suggested that perceived higher cognitive competence applied in schoolwork is associated with higher belonging to school, which in turn leads to a greater general sense of connectedness, optimism, and happiness in adolescents.**	**+ cognitive competence, optimism**	**+ academic competence**	**+ school belonging, social competence, sense of connectedness**	**Slovak**	**Slovakian**	**15**
**17**	**Singh, S., and Devender, S. (2015). Hope and mindfulness as correlates of happiness.**	**Indian Journal of Positive Psychology, 6(4), 422.**	**Oxford Happiness Questionnaire (OHQ)**	**180 students from India (Age Range = 18 to 24 years; 44% Female, 56% Male).**	**Hope and mindfulness shared a positive relationship with happiness. Hope, or a hopeful attitude, and mindfulness are significant contributors to happiness. No significant gender difference between hope, mindfulness, and happiness was found. The results revealed a positive relationship between hope, mindfulness, and happiness. Happiness is a mental state of positive emotions ranging from contentment to intense joy, which are the characteristics of well-being.**	**+ mindfulness, positive emotions, contentment, joy. Well-being**	**+ hope**		**India**	**Indian**	**18**
**18**	**Terrill, A. L., Müller, R., Jensen, M. P., Molton, I. R., Ipsen, C., and Ravesloot, C. (2015). Association between age, distress, and orientations to happiness in individuals with disabilities.**	**Rehabilitation psychology, 60(1), 27.**	**Orientations to Happiness Questionnaire (OTHQ)**	**508 Adults from the USA (Mean age = 62 years, SD = 16.25, Range = 19 to 99 years; 60% Female, 40% Male).**	**Greater distress (behavioral health) was associated with lower global happiness. Middle-aged and younger people with disabilities may be particularly affected by lower levels of happiness. Enhancing psychological strengths and resources that an individual can use increased happiness and well-being (Seligman, Steen, Park, and Peterson, 2005).**	**− distress, disabilities + enhancing psychological strengths, well-being**			**USA**	**American**	**43**
**19**	**Teshale, S. M., and Lachman, M. E. (2016). Managing daily happiness: The relationship between selection, optimization, and compensation strategies and well-being in adulthood.**	**Psychology and Aging, 31(7), 687.**	**Fordyce Emotions Questionnaire (FEQ)**	**145 Adults from the USA (Mean age = 50.53, SD = 19.17; Age Range = 22–94; 60% Female, 40% Male).**	**The greater use of selection, optimization, and compensation strategies (SOC)—elective selection (choosing a particular goal or set of goals to pursue), loss-based selection (selecting goals in the face of resource loss), optimization (enhancing or acquiring resources to achieve a goal), and compensation (reallocating resources towards another goal to maintain functioning at a specific level)—was related to greater happiness. Older and middle-aged adults showed a significant positive relationship between daily SOC usage and happiness. Baseline life satisfaction significantly predicted daily happiness. The results of this study indicate that everyday selective optimization and compensation usage are related to same-day happiness.**		**+ optimization, compensation, having set of goals to pursue, baseline life satisfaction**	**+ positive relationships**	**USA**	**American**	**46**
**20**	**Vázquez, J. J., Panadero, S., and Rivas, E. (2015). Happiness among poor women victims of intimate partner violence in Nicaragua.**	**Social work in public health, 30(1), 18–29.**	**Structured interview with illustrative figures on a 7-point scale.**	**136 Nicaraguan women (Mean age = 31.67, SD = 8.921; 100% Female).**	**Most of the women in poverty/victims of intimate partner violence (IPV) who showed an optimistic future outlook and higher feelings of social support showed greater happiness. Happiness associated with social relations. Various studies highlight the existence of a positive relationship between happiness and level of income (Andrews, 1986; Diener, Sandvik, Seidlitz and Diener, 1993; Oswald, 1997) and note that the individual’s personal financial situation may play an important role in accounting for happiness (Rojas, 2011). Social relationships and stable ties with family, friends, partners, and community are to a large extent predictors of subjective well-being and overall happiness (Diener and Seligman, 2002; Gustavsson et al., 2012). The highest percentage of happy women were those who had never filed an official complaint against their partner.**	**+ subjective well-being**	**+ optimistic outlook, level of income, personal financial situation**	**+ feelings of social support, social relations, stable family, stable friends, stable partner, community**	**Nicaragua**	**Nicaraguan**	**28**
**21**	**Warr, P. (2018). Self-employment, personal values, and varieties of happiness–unhappiness.**	**Journal of Occupational Health Psychology, 23(3), 388.**	**European Social Survey (ESS)**	**2304 Individuals (Mean age = 47.16; 30% Female, 70% Male).**	**Job satisfaction in self-employed workers vs. organizational workers related to greater happiness. Seligman (2020) introduces the notion of ‘authentic happiness’ deriving from the use of one’s personal strengths and virtues to achieve goals that have intrinsic value beyond mere pleasure, and subsequently, (Seligman, 2011) preferred the label flourishing, and parallel perspectives have used the term self-realization (Waterman, 1993) and self-validation (Warr, 2007).**		**+ job satisfaction, self-employment, organized work, personal strengths, goal achievement, intrinsic goals, self-realization**		**UK**	**British**	**104**
**22**	**Watson, G. (1930). Happiness among adult students of education.**	**Journal of Educational Psychology, 21(2), 79.**	**Happiness Questionnaire**	**338 Students from the USA (Mean age = 30).**	**Factors contributing to happiness were the enjoyment of and success in work, good health in childhood, popularity, success in dealing with people, marriage, election to offices, the love of nature, and serious hard-working living. Three major factors of unhappiness were: failure in love, the expectancy of loneliness in old age, fears, and shyness. The individual is called happy if he believes himself happier than most others of like age and sex, if he believed his prevailing moods cheerful, his spirits high, his satisfaction lasting, his days full of interesting and amusing things, his prevalent attitude described by such words as ‘enthusiastic’, ‘jolly’, ‘tranquil’, etc.**	**+ good health, − fears, shyness, enthusiasm, joy**	**+ enjoyment of work, success at work, popularity, success in dealing with people, election to office, hardworking living**	**+ marriage, love of nature, tranquility, − failure in love, loneliness in old age**	**USA**	**American**	**103**
**23**	**Xiang, Y., Wu, H., Chao, X., and Mo, L. (2016). Happiness and social stratification: A layered perspective on occupational status.**	**Social Behavior and Personality: an international journal, 44(11), 1879–1888.**	**Self-report item using 6-point Likert Scale)**	**9940 Chinese participants (Mean age = 42.69 years, SD = 14.38, Range = 15 to 64 years; 55% Female, 45% Male).**	**Higher occupational status corresponded to greater happiness. Overt occupational characteristics of power and occupational prestige and covert characteristics of justice, self-confidence, and mental health were significant as objective social factors promoting happiness. Through a survey, we found that higher occupational status corresponded to greater happiness. Our results further confirm that happiness can be stratified based on occupational status, and our findings add to understanding of the mechanism behind how occupational status affects happiness.**		**+ higher occupational status**		**China**	**Chinese**	**51**

**Table A3:** Harmony and Happiness, final count (N = 76).

						Health	Hope	Harmony	74 Unique		
Se	Study	Journal	Happiness Measure	Sample	Correlation of Happiness in Findings	Reviewers’ Agglutination of the Findings with the Three Categories Identified			Country	Culture	Quality Score (h5 Median)
**1**	**Aber, J. L., Belsky, J., Slade, A., and Crnic, K. (1999). Stability and change in mothers’ representations of their relationship with their toddlers.**	**Developmental Psychology, 35(4), 1038.**	**Parent Development Interview (PDI)**	**125 parents from the USA (Mean ages females and males = 28.4 and 30.6 years).**	**Positive mothering led to increased joy and pleasure. Stability analysis suggested a dynamic relationship between mothers’ representations of joy, pleasure, coherence, and anger.**	**+ increased joy, pleasure**		**+ positive mothering, dynamic relationship between mothers**	**USA**	**American**	**87**
**2**	**Addai, I., Opoku-Agyeman, C., and Amanfu, S. K. (2014). Exploring predictors of subjective well-being in Ghana: A micro-level study.**	**Journal of Happiness Studies, 15(4), 869–890.**	**World Values Survey (WVS)**	**1533 individuals from Ghana/Sub-Saharan Africa (Mean Age = 33.86; SD = 14.07; Age Range = 16 to 90 years; 49% Female, 51% Male).**	Personal happiness **was predicted by** economic (upper income), upper social class, social capital (freedom of choice and honesty), community engagement, perceived good health status, active religious involvement/regular attendance to religious services, **and** ethnicity/culture (Akan). **The data reveal that both happiness and life satisfaction among Ghanaians are shaped by a multitude of factors including economic, cultural, social capital, and health variables. The outcome of the study also establishes the fact that factors predicting subjective well-being (SWB) at the micro level vary in Sub-Saharan Africa contexts compared to the developed world. Collecting information about how people feel about their own life circumstances has gained political attention and acceptance in developed countries. For instance, in November 2010, the newly elected British government announced that in addition to traditional data such as income levels or fear of violent crimes, it would start measuring national happiness. The Atkinson Foundation released a Canadian Index of Well-being that focused primarily on non-monetary aspects of well-being (Brooker and Hyman 2010). SWB is portrayed as a measure that attempts to capture the overall sense of well-being, including happiness and satisfaction in life as a whole and other domains of life (see Veenhoven, 1991).**	**+ perceived good health status, health variables, subjective well-being**	**+ economic growth, increase in income, freedom of choice, economic capital, satisfaction with life**	**+ increase in social class, social capital, religious involvement, regular attendance to religious services, community engagement, active religious involvement, ethnicity, culture, − fear of violent crimes**	**Ghana**, Sub-Saharan Africa	**Ghanaian**, Sub-Sahara African	**82**
**3**	**Adjei, P. O. W., and Agyei, F. K. (2015). Biodiversity, environmental health, and human well-being: analysis of linkages and pathways.**	**Environment, Development and Sustainability, 17(5), 1085–1102.**	**Short Depression Happiness Scale (SDHS)**	**236 individuals from the UK (Age Range = 18+ years).**	**Level of ecological diversity derived from a green environment, diversity of introduced species, perceived naturalness, density of visitors, and visitors’ age (mature) showed higher levels of happiness. The paper applies happiness as a measure of well-being and examines the relationship between human well-being and the level of quality of biodiversity in the natural environment. It established that the level of ecological diversity determines the level of people’s wellness and happiness derived from a green environment. Visitors to green spaces with higher plant diversity receive higher levels of happiness. Veenhoven (2006) shares the view that when well-being and happiness are used in a broader sense, they all stand for the same thing, symbolizing good life. The well-being component expresses one’s external happiness, and the contentment conveys one’s internal happiness.**	**+ contentment**	**+ external happiness**	**+ age, ecological diversity, green environment, diversity of plant species, perceived naturalness, density of people, quality of biodiversity, ecological diversity, green spaces**	**UK**	**British**	**87**
**4**	**Aftab, M. T., Naqvi, A. A., Al-Karasneh, A. F., and Ghori, S. A. (2018). Impact of religiosity on subjective life satisfaction and perceived academic stress in undergraduate pharmacy students.**	**Journal of Pharmacy and Bioallied Sciences, 10(4), 192.**	**Subjective Happiness Scale (SHS)**	**242 students from Saudi Arabia (Mean age = 20.76 years, SD = 1.3; 61% Female, 39% Male).**	**Subjective happiness was positively correlated with non-organized religious activity and intrinsic religiosity. Subjective happiness was positively + correlated with non-organized religious activity and intrinsic religiosity (*p* < 0.01). Items related to subjective happiness investigated the degree of happiness in relation to various life events. Non-organized religious activity (NORA) had a significant (*p* < 0.01) and a moderate positive correlation with IR (+0.172) and subjective happiness (+0.182). IR was positively correlated (+0.241) with subjective happiness with statistical significance (*p* < 0.01).**			**+ intrinsic religiosity, non-organized religious activity, positive life events**	**Saudi Arabia**	**Saudi Arabian**	
**5**	**Akin, Ü. and Akin, A. (2015). Mindfulness and subjective happiness: The mediating role of coping competence.**	**Ceskoslovenska Psychologie, 59(4), 359.**	**Subjective Happiness Scale (SHS)**	**292 university students from Turkey (Age Range = 18 to 29 years).**	**Subjective happiness was predicted positively by mindfulness and coping competence. Coping competence mediated on the relationship between mindfulness and subjective happiness. The terms of subjective well-being and happiness are generally used interchangeably in the literature and refer to the same experience. On the other hand, subjective happiness is a balance of positive/negative affect and overall life satisfaction (Diener, 2000).**	**+ mindfulness, coping competence**			**Turkey**	**Turkish**	**14**
**6**	**Al-Seheel, A. Y. and Noor, N. M. (2016). Effects of an Islamic-based gratitude strategy on Muslim students’ level of happiness.**	**Mental Health, Religion and Culture, 19(7), 686–703.**	**Scale of Positive and Negative Experience (SPANE) and Satisfaction With Life Scale (SWLS)**	**60 Muslim participants from Malaysia (Mean Age = 21.85; SD = 1.22, Age Range = 19 and 25 years; 84% Female; 16% Male).**	**Practicing Islamic-based gratitude exercises (associating blessings with Allah) raised participants’ happiness levels. The results generally supported the hypothesis that practicing Islamic-based gratitude exercise would result in higher happiness levels over time and suggested that the Islamic-based gratitude is beneficial in raising participants’ happiness levels. These two terms, happiness and SWB, have been used interchangeably in the literature (McGillivray and Clarke, 2006; Reyes-García et al., 2016), but well-being is a more general term that goes beyond happiness (Anger, 2008; Dodge, Daly, Huyton, and Sanders, 2012).**			**+ practicing Islamic-based gratitude exercise**	**Malaysia**	**Malayan**	**26**
**7**	**Amutio, A., Martínez-Taboada, C., Hermosilla, D., and Delgado, L. C. (2015). Enhancing relaxation states and positive emotions in physicians through a mindfulness training program: A one-year study.**	**Psychology, Health and Medicine, 20(6), 720–731.**	**Smith Relaxation States Inventory (SRSI-3)**	**42 individuals from Spain (Mean Age = 47.31; SD = 9.42; 57% Female, 43% Male).**	**Mindfulness-based stress reduction (MBSR) led to significant improvements in happiness. Positive emotions and health have been the focus of many studies, most of them within the field of “positive psychology” (Fredrickson, 2000, 2003; Gander, Proyer, Ruch, and Wyss, 2013). Relaxation states (R-States) seem to fall into four categories: basic relaxation (sleepy, physically relaxed, disengaged, mentally relaxed, rested/refreshed), positive energy (energized, happy, thankful/loving, optimistic), core mindfulness (quiet mind, awareness, acceptance, flow), and transcendence (awe and wonder, prayerful, reverent, mystery, timeless...). These positive states are associated with one’s perception of well-being at work and increased job performance (Fredrickson, 2003)**	**+ mindfulness, stress reduction, positive energy, basic relaxation**		**+ transcendence**	**Spain**	**Spanish**	**66**
**8**	**Antonucci, T. C. and Israel, B. A. (1986). Veridicality of social support: A comparison of principal and network members’ responses.**	**Journal of Consulting and Clinical Psychology, 54(4), 432.**	**Self-Report happiness item using 3-Point Scale**	**715 participants from US (Mean Age = 77; Age Range = 18 to 95 years; 61% Female, 39% Male).**	**Veridicality (perception congruence of whether social support was provided/received) was not significantly related to happiness. Well-being measures described: life satisfaction, happiness, and negative affect.**	**+ life satisfaction**			**US**	**American**	**83**
**9**	**Bennett, D. L. and Nikolaev, B. (2017). Economic freedom and happiness inequality: Friends or foes?**	**Contemporary Economic Policy, 35(2), 373–391.**	**World Values Survey (WVS)**	**1200 participants from 100 countries (Age range = 18+).**	**Less economic freedom was negatively associated with happiness inequality. Since 1981, the World Values Survey has polled nearly 100 countries, representing almost 90 percent of the world’s population. The main sample contains data on up to 92 countries, spanning the period 1981–2012 with a total of 198 country-year observations. It found that economic freedom is negatively associated with happiness inequality. The World Database of Happiness, for instance, reports that more than 9000 happiness studies have been undertaken, about half of which have been empirical papers covering up to 164 countries (Veenhoven 2015). Studying the determinants of the distribution of happiness is relevant for several reasons. Classical economists long ago recognized that human behavior is driven not only by self-interest but also by a multitude of psychological motives such as the happiness of others.**	**+ psychological motives**	**− less economic freedom**		**100 countries representing almost 90 percent of the world’s population**	**101 countries**	**30**
**10**	**Biddle, N. (2014). Measuring and analyzing the wellbeing of Australia’s indigenous population.**	**Social Indicators Research, 116(3), 713–729.**	**National Aboriginal and Torres Strait Islander Social Survey (NATSISS)**	**300 Indigenous Australians (Age Range = 15 to 55+ years; 57% Female, 43% Male).**	**Indigenous Australians were less likely to report frequent periods of happiness and more likely to report periods of extreme sadness than the non-Indigenous population. Indigenous Australians in remote areas reported higher levels of happiness. The main finding is that, when using retrospective measures, those in remote areas report higher levels of happiness than those in non-remote areas. After undertaking a stepwise regression, the Indigenous status coefficient was statistically significant in the happiness estimations at the 5% level of significance after controlling for age, sex, country of birth, marital status, family type, and level of education.**			**+ indigenous culture**	**Australia**	**Australian**	**77**
**11**	**Binion, G. and Zalewski, M. (2018). Maternal emotion dysregulation and the functional organization of preschoolers’ emotional expressions and regulatory behaviors.**	**Emotion, 18(3), 386.**	**Locked Boxed Task (Laboratory Temperament Assessment Battery)**	**68 mothers and their 3–4-year-old children from US (Mean Age = 48, SD = 7.6).**	**Maternal emotion dysregulation was associated with children displaying more sadness. Maternal non-supportive emotion socialization responses were associated with children engaging in more defiant behaviors throughout the task and using less problem solving in the context of happiness.**	**+ maternal emotion**		**+ socialization**	**US**	**American**	**79**
**12**	**Birchler, G. R. and Webb, L. J. (1977). Discriminating interaction behaviors in happy and unhappy marriages.**	**Journal of Consulting and Clinical Psychology, 45(3), 494.**	**Areas of Change Questionnaire (ACQ)**	**100 married couples from the US.**	**Unhappily married couples showed a deficit in problem solving, more unresolved problems, less involvement with one another in both elective free-time activities and less shared sexuality. Unhappily, relative to happily, married couples would show a deficit in problem solving by indicating significantly more unresolved problems.**			**− unhappily married couples, less involvement with one another, less shared sexuality**	**US**	**American**	**83**
**13**	**Blekesaune, M. (2018). Is cohabitation as good as marriage for people’s subjective well-being? longitudinal evidence on happiness and life satisfaction in the British household panel survey.**	**Journal of Happiness Studies, 19(2), 505–520.**	**General Health Questionnaire (GHQ)**	**10,300 individuals from Great Britain (Age Range = 20 to 64 years).**	**Entering cohabitation is as beneficial as entering marriage for people’s happiness. The hedonic adaptation theory (Brickman and Campbell, 1971) and set-point theory (Diener et al., 2006) suggest that happiness is an emotional state that is linked to one’s physiological reactions to life events, whereas satisfaction is a cognitive evaluation that also depends on social comparisons with other important reference groups as well as the individual’s desires, expectations, and hopes. These findings indicate that cohabitation provides similar benefits to marriage regarding happiness but not how previously never-married individuals view their overall satisfaction with their lives. Subjective well-being is investigated as changes in happiness and life satisfaction associated with entering cohabitation and marriage among 1436 never married and 1539 previously married individuals who were observed in up to 12 waves in the British Household Panel Survey (BHPS), from about 1997 to 2009.**			**+ cohabitation, marriage**	**Great Britain (Ireland, Scotland, England)**	**British (Irish, Scottish, English)**	**82**
**14**	**Brailovskaia, J. and Margraf, J. (2016). Comparing Facebook users and Facebook non-users: Relationship between personality traits and mental health variables–an exploratory study.**	**PLoS ONE, 11(12), 1–17.**	**Subjective Happiness Scale (SHS)**	**790 participants from Germany (Mean Age = 23.42; SD = 5.02, Age Range = 17–5; 64% Female, 36% Male).**	**Facebook users had significantly higher subjective happiness. Extraversion, self-esteem, happiness, life satisfaction, resilience, and social support, on the one hand, and depression, anxiety, and stress symptoms, on the other hand, are negatively correlated. Positive mental health variables, such as happiness, resilience, life satisfaction, and social support. Positive (subjective happiness, resilience, life satisfaction, social support) variables of mental health.**	**+ extraversion, life satisfaction, resilience, − depression, anxiety, stress**	**+ self-esteem**	**+ Facebook users, social support**	**Germany**	**German**	**278**
**15**	**Calvo, R., Arcaya, M., Baum, C. F., Lowe, S. R., and Waters, M. C. (2015). Happily, ever after? Pre-and-post disaster determinants of happiness among survivors of Hurricane Katrina.**	**Journal of Happiness Studies, 16(2), 427–442.**	**Self-report item using 4-categories scale**	**491 women survivors from US (Mean age = 25.2 years, SD = 4.6; 84% Female; 16% Male).**	**Happiness significantly decreased post-disaster. Living alone, low perceived social support, exposure to hurricane stressors (shortage of water/food, etc.), or the death of a loved one led to decreased happiness post-disaster. The study investigated pre- to post-disaster changes in the happiness of 491 women affected by Hurricane Katrina and identified factors that were associated with the survivors’ happiness after the storm. They found that happiness significantly decreased from pre-disaster to 1-year post-disaster but there were no significant differences in happiness between the pre-disaster and 4 years post-disaster assessments. Happiness is only a partial representation of subjective well-being (Raibley 2012)—a complex concept that lacks universal definition, but which is often understood as a personal assessment of one’s life in general composed of a long-term cognitive dimension and a temporal affective dimension (Diener, 1984; Diener et al., 1999; Lucas et al., 1996).**			**− living alone, low social support, death of a loved one, exposure to water/food shortages**	**US**	**American**	**82**
**16**	**Chiu, S. W. K. and Wong, K. T. W. (2018). Happiness of Hong Kong youth from 2000 to 2014: empirical evidence on the differential impact of socioeconomic conditions on youth versus other age groups.**	**Journal of Youth Studies, 21(3), 253–271.**	**Hong Kong Social Image Survey (self-report happiness item using 4-Point Scale)**	**11,184 participants from China (Age Range = 31 to 51+ years).**	**Reduced happiness is more significant for the youth than for older people. Rapid economic growth and the rise in the price of housing have made older people happier than the youth since the early 2010s. The change in happiness through the life course is believed to reflect a process of role transition and adaptation to circumstances. Happiness is derived from a sense of accomplishment in one’s life.**		**+ rapid economic growth, rise in the price of housing, sense of accomplishment in life**		**China**	**Chinese**	**47**
**17**	**Chouhan, M. and Gupta, S. (2015). Effect of resilience on well-being of Kashmiri pandit migrants.**	**Indian Journal of Health and Wellbeing, 6(6), 575.**	**Friedman Well-Being Scale (FWBS)**	**88 Kashmiri migrants from India (Age Range = 35 to 50 years; 50% Female, 50% Male).**	**Higher resilience was associated with greater joviality and happiness. Males showed greater joviality and happiness than females, noting gender effects. Gender significantly affected the three components of well-being (joviality, happiness, and sociability).**	**+ higher resilience, greater joviality**		**+ sociability**	**India**	**Indian**	
**18**	**de Souto Barreto, P. (2014). Direct and indirect relationships between physical activity and happiness levels among older adults: a cross-sectional study. Aging & mental health, 18(7), 861–868.**	**Aging and Mental Health, 18(7), 861–868.**	**Single item measure of happiness—self-report questionnaire.**	**323 participants from France (Mean age = 72.9, SD = 7.5; Age Range = 63 to 96 years; 66% Female; 34% Male).**	**Physical activity was associated with higher levels of happiness. Higher social functioning mediated the indirect relationship between physical activity and greater happiness. A structural equation modeling (SEM) showed an indirect association between PA and happiness, which was mediated by participants’ health status and social functioning.**	**+ physical activity**		**+ higher social functioning**	**France**	**French**	**73**
**19**	**Delhey, J. and Dragolov, G. (2016). Happier together. Social cohesion and subjective well-being in Europe.**	**International Journal of Psychology, 51(3), 163–176.**	**European Quality of Life Survey.**	**34,891 participants from 34 countries (Age Range = 18 and above).**	**Pos+ with Facebook users, community. Europeans are indeed happier and psychologically healthier in more cohesive societies. Life evaluation is a hedonic orientation defined as happiness in terms of life satisfaction (Veenhoven, 2012). Post-materialization of happiness—the idea of a relative shift in happiness recipes, away from economic concerns towards non-economic ones (Delhey, 2010; Wenzel and Inglehart, 2010). Subsume happiness and life satisfaction under life evaluation by taking their arithmetic mean for everyone. According to the findings from the qualitative stage of the research, peaceful and happy living means having comfort and positive feelings and not having negative feelings.**	**+ life satisfaction, not having negative feelings**	**+ post-materialization**	**+ Facebook users, community, cohesive societies, peaceful living**	**34 countries**	**35 countries**	**45**
**20**	**Demirci, İ. and Ekşi, H. (2018). Keep calm and be happy: A mixed method study from character strengths to well-being.**	**Educational Sciences: Theory and Practice, 18(2).**	**Happy Living Semi-Structured Interview**	**900 participants from Turkey (Mean Age = 30.46; SD = 12.94; Age Range = 18 to 75 years; 61% Female, 39% Male).**	**Sufficiency in relationships and trust, personal virtues, social virtues, acceptance, spirituality, developmental strength of problems, optimism, nature, health, and economics, as well as activities and superficial solutions, constituted sources of happiness. Happiness was positively correlated with the characteristics of tolerance, helpfulness, beliefs, spirituality, responsibility, purposefulness, worthiness, trust, and reliability. The research has found peace and happiness to positively correlate with the characteristics of tolerance, helpfulness, beliefs and spirituality, responsibility, purposefulness, worthiness, trust, and reliability.**	**+ optimism, health**	**+ economics**	**+ sufficiency in relationships, trust, personal virtues, social virtues, acceptance, spirituality, nature,**	**Turkey**	**Turkish**	**47**
**21**	**Edwards-Leeper, L., Feldman, H. A., Lash, B. R., Shumer, D. E., and Tishelman, A. C. (2017). Psychological profile of the first sample of transgender youth presenting for medical intervention in a US pediatric gender center.**	**Psychology of Sexual Orientation and Gender Diversity, 4(3), 374.**	**Archival Chart Review**	**56 gender dysphoric youth from the US (Age Range = 8.9 to 17.9 years).**	**The first group of transgender youths to seek medical interventions in the first U.S. interdisciplinary pediatric gender clinic: older youths experienced decreased happiness than the younger patients. Findings suggest youths with gender dysphoria may experience a progressively lower quality of life, less happiness, and lower self-esteem as they get older**	**− transgender youth seeking medical intervention, gender dysphoria**	**− lower self-esteem**		**US**	**American**	**46**
**22**	**El Zein, R., Kobaladze, M., and Vacharadze, K. (2016). Happiness and Well-Being as Seen by Children and Young People in Georgia.**	**Education Sciences and Psychology, 42(5).**	**Individual and Group Interviews**	**20 participants (Age Range = 10 to 19 years).**	**Children related the health of their parents and doing well in school to happiness. Interviews and focus groups conducted by CRRC-Georgia in November 2014 focuses on the views of children** ** and young people themselves—their own experiences and understanding of what well-being and happiness mean. The findings of the study are largely not surprising, with** **children relating the health of their parents and doing well in school to happiness and teenagers relating increased independence and opportunity as intrinsic to well-being. Georgia’s research noted differences in definitions of happiness and well-being between children (aged 10–12) and teenagers (aged 15–18)**		**+ doing well in school, increased independence**	**+ health of parents**	**Georgia**	**Georgian**	**16**
**23**	**Elliot, M., Cullen, M., and Calitz, A. P. (2018). Happiness among South African private sector physiotherapists.**	**The South African Journal of Physiotherapy, 74(1).**	**Oxford Happiness Questionnaire (OHQ)**	**395 respondents from South Africa (Age Range = 20 to 40 years; 88% Female; 12% Male).**	**Influence, social relations, life balance, optimism, work, and leisure were all positively associated with happiness levels. The happiness of people can affect their daily functioning and work performance. Although researchers and laypeople often define happiness as life satisfaction or a sense of well-being, studies also define happiness as positive subjective experiences (Delle Fave et al. 2016; Joshanloo and Weijers 2014; Scorsolini-Comin and Dos Santos 2010; Wren-Lewis 2014)**	**+ life balance, optimism**	**+ work**	**+ social relations, influence, leisure**	**South Africa**	**South African**	
**24**	**Fleischmann, F. and Verkuyten, M. (2016). Dual identity among immigrants: Comparing different conceptualizations, their measurements, and implications.**	**Cultural Diversity and Ethnic Minority Psychology, 22(2), 151.**	**Self-report happiness item using 5-Point Scale**	**70,000 immigrants from the Netherlands (Age Range = 25–45; 51% Female, 49% Male).**	**National identification, ethnic identification, self-identification, strict identity duality, perceived acceptance, and feeling at home in NL were significantly positively associated with happiness. Perceived group and personal discrimination were significantly negatively associated with happiness. A one-item measure assessed how happy participants are, ranging from 1—very happy to 5—unhappy. The research recorded higher scores indicated greater happiness. It investigated how feeling at home in the Netherlands, and happiness are related. It is crucial for happiness that both national and ethnic identity are considered of equal importance. Higher national and ethnic identification are both positively related to happiness.**			**+ national identification, ethnic identification, self-identification, strict identity duality, perceived acceptance, dealing at home, − group discrimination, personal discrimination**	**Netherlands**	**Netherlander**	**48**
**25**	**Forooshany, S. H. A., Yazdkhasti, F., Hajataghaie, S. S., and Esfahani, M. H. N. (2014). Infertile individuals’ marital relationship status, happiness, and mental health: a causal Model.**	**International Journal of Fertility and Sterility, 8(3), 315.**	**Oxford Happiness Questionnaire (OHQ)**	**155 participants from Iran (66% Female; 34% Male).**	**Fewer difficulties in marital relationship status were positively related to happiness. A causal model of the relation between marital relationship status, happiness, and mental health. Marital relationship status was directly related to happiness (*p*<0.05), and happiness was directly related to mental health, (*p*<0.05). Happiness, which is an essential dimension of life and related to functioning and success, generally is considered to comprise three main components—frequency and degree of positive affect or joy; absence of negative feelings, such as depression or anxiety; and the average level of satisfaction over a period.**			**+ less difficulty in marital relationship**	**Iran**	**Iranian**	
**26**	**Frash, R. E., Jr., Blose, J. E., Norman, W. C., and Patience, M. (2016). Healthy parks, happy people: An exploratory study of a county park system.**	**Journal of Park and Recreation Administration, 34(1).**	**Self-report happiness item using 7-Point Scale**	**324 participants from the USA (Median Age = 39; 60% Female, 40% Male).**	**Park visitation, greater diversity of park activities during one visit, and pleasure with park operations were factors that stimulated happiness. Happiness is a quality that nearly all people naturally desire. Happiness has been shown to have a host of positive features, including enhanced mental and physical health, more satisfactory relationships, increased earning potential, and even longer life. Park activities and happiness are aspects of leisure and subjective well-being. There was a positive relationship between park satisfaction and happiness**			**+ park visitation, greater diversity of park activities, pleased with park operations**	**USA**	**American**	**28**
**27**	**Frutos, A. M., and Merrill, R. M. (2017). Viewing explicit sexual movies was related to less happiness in marriage.**	**Sexuality and Culture, 21(4), 1062–1082.**	**General Social Survey (GSS)**	**11,372 participants from the USA (Age Range = 18 and above; 55% Female, 45% Male).**	**Viewing explicit sexual movies was related to less happiness in marriage. Viewing explicit sexual movies was related to less happiness in marriage. Male viewers experienced a reduction in the positive effect of the frequency of sex on happiness (Doran and Price 2014).**	**− viewing explicit sexual movies**			**USA**	**American**	**37**
**28**	**Fu, R. and Noguchi, H. (2016). Does marriage make us healthier? Inter-country comparative evidence from China, Japan, and Korea.**	**PLoS ONE, 11(2).**	**2010 East Asian Social Survey.**	**7938 participants from China, Japan, and Korea (Age Range = 18 to 65+ years).**	**A higher balanced marriage with intra-couple education (both husband and wife are well-educated) demonstrated higher happiness. Couples tend to be happier when both the husband and the wife are well educated. The coefficients of happiness are almost always significantly positive in all countries (9.2% in China, 23.3% in Japan, and 13.6% in Korea), implying better happiness levels for married people under 50.**			**+ higher balanced marriage, marriage, intra-couple education**	**China, Japan, and Korea**	**Chinese, Japanese, and Korean**	**278**
**29**	**Graham, C. and Pozuelo, J. R. (2016). Happiness, Stress, and Age: How the U-Curve Varies across People and Places.**	**Journal of Population Economics, 30, 1–29.**	**World Happiness Report (2015)**	**9000 participants from 44 countries (Age Range = 15+).**	**A U-shaped relationship was found between age and happiness (i.e., middle-aged had less happiness). The timing of the turn varies depending on the average country-level happiness and on individuals’ positions in the well-being distribution. There is now much evidence for a remarkably consistent relationship between age and happiness. The U-shaped relationship between age and happiness held in 44 of the 46 countries. Measures of life satisfaction, happiness, reported mental illness, and/or daily moods and experiences—ranging from contentment to stress and anger—help us understand a range of behaviors, as well as their welfare benefits or costs, across individuals, countries, and generations. Numerous studies have found recurrent patterns between happiness and life satisfaction (whereas the terms are often used interchangeably, the latter is a better-specified question) and important experiences such as employment, marital status, and/or earnings.**	**− mental illness, stress, anger**	**+ individuals’ position, employment, earnings**	**− marital status, middle age**	**Albania, Argentina, Australia, Belgium, Bosnia, Brazil, Bulgaria, Canada, Chile, China, Colombia, Croatia, Cyprus, Czech Republic, Denmark, Estonia, Finland, France, Germany, Greece, Hungary, India, Ireland, Italy, Kosovo, Latvia, Lithuania. Macedonia, Mexico, Montenegro, Netherlands, Peru, Poland, Portugal, Romania, Russia, Serbia, Slovakia, Slovenia, South Africa, Spain, Sweden, UK, US, Venezuela**	**Albanian, Argentinian, Australian, Belgium, Bosnian, Brazilian, Bulgarian, Canadian, Chilean, Chinese, Colombian, Croatian, Cyprus, Czech, Denmark, Estonian, Finish, French, German, Greek, Hungarian, Indian, Irish, Italian, Kosovan, Latvian, Lithuanian. Macedonian, Mexican, Montenegrin, Netherlands, Peru, Polish, Portuguese, Romanian, Russian, Serbian, Slovakian, Slovenian, South African, Spanish, Swedish, English, American, Venezuelan**	**55**
**30**	**Gudmundsdóttir, D. G., Ásgeirsdóttir, B. B., Huppert, F. A., Sigfúsdóttir, I. D., Valdimarsdóttir, U. A., and Hauksdóttir, A. (2016). How does the economic crisis influence adolescents’ happiness? Population-based surveys in Iceland in 2000–2010.**	**Journal of Happiness Studies, 17(3), 1219–1234.**	**Self-report item using 4-point Likert Scale)**	**28 adolescents from Iceland (Age Range = 14 to 15 years).**	**Gender, age, family structure, parents’ education, time spent with parents, and emotional support from parents altogether explained 13% of the happiness variance. Emotional support from parents together with time spent with parents had the largest positive influence on happiness. The results indicated that happiness increased by 5% in the adolescent population from 2000 to 2010 despite the economic crisis. The association between socio-demographic factors and happiness was explored using multiple linear regression analyses. Bentham defined happiness in 1789 as ‘‘the sum of pleasures and pains’’ (Bentham 1789/1996) which can be interpreted in the way that happiness is not only about positive feelings, but also about how well one copes with obstacles or pain.**	**+ positive feeling, coping with obstacles**		**+ gender, age, family structure, parent’s education, time spent with parents, emotional support from parents, socio-demographic factors**	**Iceland**	**Icelander**	**82**
**31**	**Haj-Yahia, M. M. (2002). The impact of wife abuse on marital relations as revealed by the second Palestinian National Survey on Violence Against Women.**	**Journal of Family Psychology, 16(3), 273.**	**Marital Happiness Scale (MHS)**	**1686 women from Palestine (Mean age = 31.5 years, SD = 9.1, Range = 17 to 69 years; 100% Female).**	**Abused wives (physically abused, psychologically abused, sexually abused, and/or economically abused) expressed lower levels of both harmony and happiness. A survey (conducted from June to August 1995) also investigated some psychological effects of such experiences (i.e., low self-esteem, depression, and anxiety; Haj-Yahia, 2000a) as well as some marital effects (i.e., commitment, communication, satisfaction, affection, harmony, and happiness). Palestinian women were hypothesized to be psychologically, physically, sexually, and economically abused (as the main predictor variables of the study); the lower their levels of marital commitment, the more negative were their patterns of communication with their husbands, and the lower were their levels of marital satisfaction, affection, happiness, and harmony (as the main criterion variables of the study).**	**− physically abused, psychologically abused, sexually abused, depression, anxiety**	**− economically abused, low self-esteem**	**+ marital commitment, − lower harmony, negative communication with husbands**	**Palestine**	**Palestinian**	**52**
**32**	**Hart, E. A. C., Lakerveld, J., McKee, M., Oppert, J. M., Rutter, H., Charreire, H., ... and Brug, J. (2018). Contextual correlates of happiness in European adults.**	**PLoS ONE, 13(1).**	**Self-report happiness item using 5-Point Scale**	**6037 Participants from Europe (Mean age = 52.1, SD = 16.3; 55% Female; 45% Male).**	**Neighborhoods with higher levels of aesthetics, more water, more green space, perceived as safer, and greater function were associated with greater happiness. The perceived absence of air pollution was associated with higher levels of happiness in more highly educated participants. Individuals with a larger social network, more social cohesion, and trusted neighbors showed greater happiness. The association between social networks and happiness was stronger in men than women. Associations of 14 physical and social environmental characteristics with happiness were analyzed using multivariable multinomial regression analyses with clustered standard errors. Living in neighborhoods with higher levels of aesthetics and more water and green space was associated with being very happy. A commonly used definition of happiness is “the degree to which an individual judges the overall quality of his/her life as a whole favorably”.**			**+ neighborhoods with high aesthetics, more water, green spaces, safer spaces, absence of air pollution, larger social network, more social cohesion, trusted neighbors, association between social networks**	**Europe**	**European**	**278**
**33**	**Herbst, C. M. and Lucio, J. (2016). Happy in the hood? The impact of residential segregation on self-reported happiness.**	**Journal of Regional Science, 56(3), 494–521.**	**National Survey of Families and Households (NSFH)**	**14,334 participants from the USA (Mean Age = 44.54, SD = 16.74, Age Range = 14+ years; 60% Female, 40% Male).**	**Segregation is associated with a reduction in happiness among the Black population (i.e., members of the Black population are found to be happier in more segregated metropolitan areas). This paper explored the relationship between residential segregation and self-reported happiness. Increased segregation is associated with a reduction in happiness among the Black population. Definitions of happiness typically focus on the cognitive dimensions of how people feel about their lives; the well-known connection between self-reported happiness and physical health suggests that the results from this study are potentially relevant to a broad set of health outcomes (Diener and Seligman, 2004; Frey and Stutzer, 2002; Kahneman and Deaton, 2011).**			**− increased segregation in the black population**	**USA**	**American**	**42**
**34**	**Ho, H. C., Mui, M., Wan, A., Ng, Y. L., Stewart, S. M., Yew, C., ... and Chan, S. S. (2016). Happy Family Kitchen: A community-based research for enhancing family communication and well-being in Hong Kong.**	**Journal of Family Psychology, 30(6), 752.**	**Subjective Happiness Scale (SHS), and Single Item Indicator self-report scale (happiness)**	**1419 participants from China (Age Range = 6 to 65+ years; 65% Female, 35% Male).**	**Community-based family intervention showed that family communication, family well-being, and gratitude intervention improved family happiness and subjective happiness. Community-based family intervention, derived from a positive psychology framework, can improve family communication, family well-being, and subjective happiness. The positive emotion of happiness among married couples is positively associated with the frequency of marital interaction, such as eating, shopping, and visiting friends together (Zuo, 1992). The literature on positive psychology and the theoretical framework encompassed gratitude, flow, happiness, health, and savoring (Soong et al., 2015).**			**+ family communication, family well-being, gratitude intervention**	**China**	**Chinese**	**52**
**35**	**Ho, H. C., Mui, M., Wan, A., Stewart, S. M., Yew, C., Lam, T. H., and Chan, S. S. (2017). Happy Family Kitchen: Behavioral outcomes of a brief community-based family intervention in Hong Kong.**	**Journal of Child and Family Studies, 26(10), 2852–2864.**	**Subjective Happiness Scale (SHS), and single item indicator self-report scale (happiness)**	**936 participants from China (Age Range = 35 to 40 years; 68% Female, 32% Male).**	**Gratitude, flow, and happiness interventions were effective in increasing happiness, happy memories, and happiness behaviors. Family communication and eating with family were significantly positively associated with family happiness. Using a one-group pre-test and repeated post-test design over a 12-week period, the results showed that the overall intervention program improved family communication quality, family health, happiness and harmony, and subjective happiness. The gratitude and savoring interventions improved all the outcome measures, whereas the happiness intervention improved family communication quality, family health, family happiness, and subjective happiness but not family harmony. The flow and health interventions improved family communication quality and subjective happiness, respectively.**	**+ happy memory**		**+ gratitude, family communication quality, eating with family, family happiness, family health**	**China**	**Chinese**	**71**
**36**	**Jindal, Y. K. and Jain, M. (2017). Comparison between father and mother of differently abled children on hope and happiness.**	**Indian Journal of Health and Wellbeing, 8(10).**	**Oxford Happiness Questionnaire (OHQ)**	**40 parents from India (Age Range 30–45 years; 42% Female; 58% Male).**	**Statistically, no significant difference was found between fathers and mothers of differently abled children on hope and happiness. Happiness is generally believed to be determined in part by one’s unique personality, set point, and general situation and is especially influenced by frequent, small, positive events (Argyle, 2001; Diener and Emmons, 1984; Veenhoven, 1094). Hope is defined as a set of positive expectations and wishes about the future that may condition a person’s present affective state such as happiness.**			**+ personality, general situation, positive events**	**India**	**Indian**	**36**
**37**	**Kasymova, S. and Billings, D. L. (2018). Meanings of fatherhood in urban Tajikistan. Psychology of Men and Masculinity, 19(4), 635.**	**Psychology of Men and Masculinity © 2017 American Psychological Association** **2018, Vol. 19, No. 4, 635–644**	**Mixed-methods approach, which incorporated convenience and snowball sampling techniques.**	**Qualitative interviews with 30 fathers with children ages 0 to 19 years old who lived in urban areas of Tajikistan.**	**Most respondents commented on the joy, happiness, prosperity, inspiration, and support fatherhood brought to them. (n = 11) described their children as a source of joy, happiness, prosperity, inspiration, and support.**	**+ joy, inspiration**	**+ prosperity**		**Tajikistan**	**Tajikistani**	**49**
**38**	**Keng, S. H. and Wu, S. Y. (2014). Living happily ever after? The effect of Taiwan’s National Health Insurance on the happiness of the elderly.**	**Journal of Happiness Studies, 15(4), 783–808.**	**Center for Epidemiological Studies Depression Scale (CES-D)**	**4049 participants from Taiwan (Age Range = 60 and over).**	**National health insurance significantly increased happiness. Taiwan’s national health insurance (NHI) in 1995 created a quasi-experiment that permits us to draw causal inferences for the effect of the NHI on happiness and life satisfaction. The results show that the NHI has a significant effect on happiness and life satisfaction. Happiness has long been a main theme in psychology and sociology; it is only recently that economists have linked psychological studies to economics (Easterlin 1974). Among all identified determinants of psychological well-being, income is the leading factor that much research that has been undertaken (for reviews, see Clark et al., 2008).**		**+ national insurance, income**		**Taiwan**	**Taiwanese**	**82**
**39**	**Krause, N., Pargament, K. I., Hill, P. C., and Ironson, G. (2018). Assessing the role of race/ethnicity in the relationships among spiritual struggles, health, and well-being.**	**American Journal of Orthopsychiatry, 88(2), 132.**	**Subjective Happiness Scale (SHS)**	**3010 participants from US (Mean age = 46.4 years, SD = 17.2, Range = 18 to 65+ years; 59% Female; 41% Male; 41% White, 14% Hispanic; 12% Black).**	**More spiritual struggles associated with less happiness. The Black population is less vulnerable to the effects of spiritual struggles on happiness than the White and Hispanic populations. The relationship between spiritual struggles, health, and well-being varies across racial/ethnic groups. Findings from the current study suggest that when spiritual struggles arise, the Black population experiences fewer symptoms of physical illness and less anxiety, and they tend to be happier than White or Hispanic people.**			**− more spiritual struggles**	**US**	**American**	**54**
**40**	**Lambert, M., Fleming, T., Ameratunga, S., Robinson, E., Crengle, S., Sheridan, J., ... and Merry, S. (2014). Looking on the bright side: An assessment of factors associated with adolescents’ happiness.**	**Advances in Mental Health, 12(2), 101–109.**	**WHO-5 Well-Being Index (WHO-5)**	**8679 secondary school students from New Zealand (Age Range = 13 to 17+ years; 46% Female; 54% Male).**	**Happiness was positively associated with good connections with family, friends, and school, regular exercise, and meals with family. Happiness was negatively associated with witnessing yelling and the hitting of children and adults at home, discrimination, frequent marijuana use, sexual abuse, frequent alcohol use, and having a long-term health condition. Determines possible factors which may be associated with happiness among New Zealand adolescents. Happiness was measured using the WHO well-being index. Protective factors for happiness included family, school, and peer connection, as well as family meals, exercise, and belonging to a cultural group. Happiness was positively associated with good connections with family, friends, and school, regular exercise, and meals with family. The measurement of life satisfaction as a concept is often used as a proxy to indicate happiness and quality of life (Biswas-Diener and Tamir, 2004).**	**+ regular exercise, − frequent marijuana use, sexual abuse, frequent alcohol use, having a long-term health condition**		**+ good connections with family, friends, school, meals with family, belonging to a cultural group, − yelling and hitting of children**	**New Zealand**	**New Zealander**	**21**
**41**	**Le, T. N., Lai, M. H., and Wallen, J. (2009). Multiculturalism and subjective happiness as mediated by cultural and relational variables.**	**Cultural Diversity and Ethnic Minority Psychology, 15(3), 303.**	**Subjective Happiness Scale (SHS)**	**338 participants from the USA (Mean Age = 12.5, Age range = 11–15 years; 47% Female, 53% Male; 50% Asian, 14% African American, 37% Hispanic, 2% Others).**	**A positive relation between perceived school multiculturalism and subjective happiness with full mediation by ethnocultural empathy for African Americans, Asians, males, and females. School multiculturalism was also predictive of ethnocultural empathy for Hispanics; ethnocultural empathy, in turn, was not significantly predictive of subjective happiness.**			**+ perceived multiculturism, − ethnocultural empathy**	**USA**	**Asian, African American, Hispanic, others**	**56**
**42**	**Levin, J. (2013). Religious behavior, health, and well-being among Israeli Jews: Findings from the European Social Survey.**	**Psychology of Religion and Spirituality, 5(4), 272.**	**Self-report happiness item using 10-Point Scale**	**1849 participants from Israel (Age Range = 15+).**	**Synagogue attendance and prayer were associated with greater happiness. Synagogue attendance is associated with greater happiness only, whereas prayer is associated with greater happiness and life satisfaction and higher scores on the well-being scale. indicators of both global subjective and functional health and on the indicators of the affective, cognitive, and somatic dimensions of psychological well-being, by way of respective measures of happiness, and life satisfaction.**	**+ functional health, psychological well-being, life satisfaction**		**+ synagogue attendance, prayer**	**Israel**	**Israeli**	**48**
**43**	**Li, C. H. and Tsai, M. C. (2014). Is the easy life always the happiest? Examining the association of convenience and well-being in Taiwan.**	**Social Indicators Research, 117(3), 673–688.**	**Taiwan Social Change Survey (TSCS)**	**1680 participants from Taiwan (Mean Age = 44.2, SD = 16.5; Age Range = 19+ years; 50% Females, 50% Males).**	**Better air quality/less pollution and quietness in the neighborhood enhanced happiness. Neighborhood amenities correlate positively with oft-used measures of well-being, such as life satisfaction and happiness.**			**+ better air quality, less pollution, quietness in the neighborhood**	**Taiwan**	**Taiwanese**	**77**
**44**	**Li, Y., Johnson, B. D., and Jenkins-Guarnieri, M. A. (2013). Sexual identity development and subjective well-being among Chinese lesbians.**	**International Perspectives in Psychology: Research, Practice, Consultation, 2(4), 242.**	**Depression–Happiness Scale**	**439 Chinese lesbians (Age Range: 18 to 42; 100% Female).**	**There was no significant relationship found between the six different sexual identity developmental stages and happiness. The subjective well-being (SWB), including satisfaction with life, hope, and happiness, was assessed in relation to stage of sexual identity development for Chinese lesbians ages 18 to 42. Women in committed relationships reported more life satisfaction and more disclosure of their sexual identity to family members.**			**+ sexual identity**	**China**	**Chinese**	**18**
**45**	**Lyu, J., Mao, Z., and Hu, L. (2018). Cruise experience and its contribution to subjective well-being: A case of Chinese tourists.**	**International Journal of Tourism Research, 20(2), 225–235.**	**Positive And Negative Affect Schedule (PANAS)**	**18 participants from China (Age Range = 24 to 65 years; 67% Female, 33% Male).**	**Travel experience (cruise tourism) created short-term happiness through emotional and relational experiences. Happiness from cruise travel is created mainly through emotional and relational experiences. SWB emphasizes the subjective nature of happiness, such as emotion and mood, rather than real-life objective standards, such as income and education level, placing individuals to be the single best judges of their own happiness (Kesebir and Diener, 2008).**	**+ emotional experience**	**+ travel experience, income, education level**	**+ relational experience**	**China**	**Chinese**	**58**
**46**	**Mehrdadi, A., Sadeghian, S., Direkvand-Moghadam, A., and Hashemian, A. (2016). Factors affecting happiness: a cross-sectional study in the Iranian youth.**	**Journal of Clinical and Diagnostic Research: JCDR, 10 (5).**	**Oxford Happiness Inventory (OHI)**	**500 participants from Iran (Mean age = 20 years, SD = 2.18, Range = 16 to 90 years; 46% Female; 54% Male).**	**Living in urban vs. rural areas, regular physical activity, and government employment associated with greater happiness. Differences in gender, marital status, and education level had no significant associations with happiness scores. There was not a significant relationship between gender, marital status, and education level with happiness scores among the participants. Concepts such as self-satisfaction, life satisfaction, and well-being are interest criteria of the World Health Organization in the definition of mental health. These components are associated with positive emotions such as joy, peace, and happiness.**	**+ regular physical activity, life satisfaction, well-being, joy**	**+ government employment, education**	**+ peace**	**Iran**	**Iranian**	
**47**	**Monin, J. K., Poulin, M. J., Brown, S. L., and Langa, K. M. (2017). Spouses’ daily feelings of appreciation and self-reported well-being. +B34:B35**	**Health Psychology, 36(12), 1135.**	**Positive affect index self-report measure**	**73 spouses from US (Mean age = 65.78, SD = 5.83; Age Range = 54 to 81years; 56% Female, 44% Male).**	**Perceived help from a spouse increased partners’ happiness, i.e., when spouses believe their help is appreciated in daily life by their partners, they feel happier. A study showed that active help was associated with more positive affect for spouses when they perceived the help increased their partner’s happiness and improved their partner’s well-being. Helping predicted greater positive affect for above-mean perceived levels of increasing partner happiness (B = 0.18, 95% CI [0.11, 0.25], φ = 0.17, *p* < 0.001) and improving partner well-being (B = 0.20, 95% CI [0.12, 0.28], φ = 0.16, *p* < 0.001).**			**+ perceived help from spouse**	**US**	**American**	**74**
**48**	**Moreno, R. L., Godoy-Inquired, D., Vazquez Perez, M. L., García, A. P., Araque Serrano, F., and Godoy Garcia, J. F. (2014). Multidimensional psychosocial profiles in the elderly and happiness: a cluster-based identification.**	**Aging and Mental Health, 18(4), 489–503.**	**Happiness Scale (HS)**	**154 participants from Spain (Mean Age = 77.4, SD = 8.03; Age Range = 65 to 96 years; 50% Female, 50% Male).**	**Highly successful and home-dwelling elders demonstrated significantly higher happiness. Highly successful elders demonstrated significantly higher happiness, positive affect, affect balance, and life satisfaction. Establishing psychosocial, changeable variables with an influence on happiness allows for a focused intervention on these personal resources to protect or enhance SWB in the elderly.**			**+ home-dwelling elders**	**Spain**	**Spanish**	**73**
**49**	**Morgan, J., Robinson, O., and Thompson, T. (2015). Happiness and age in European adults: The moderating role of gross domestic product per capita.**	**Psychology and Aging, 30(3), 544.**	**European Social Survey (ESS)**	**46,301 individuals from Europe (Age Range = 20 to 79 years).**	**Eudaimonia and hedonic happiness remained relatively stable across the lifespan only in the most affluent nations; in poorer nations, there was a fluctuating or steady age-associated decline in happiness. It was found that eudaimonia and hedonic happiness remained relatively stable across the lifespan only in the most affluent nations; in poorer nations, there was either a fluctuating or steady age-associated decline. Hedonic happiness is defined as the subjective experience of pleasure and satisfaction and the absence of pain or negative feelings (Deci and Ryan, 2006). In contrast, eudaimonia happiness is concerned with optimal experience, positive relationships, a sense of purpose, meaning, and a feeling of growth, and has been operationalized by psychologists as ‘flourishing’ (Diener et al., 2010).**	**+ pleasure, satisfaction**	**+ sense of purpose, meaning, feeling of growth**	**+ positive relationships**	**Europe**	**European**	**46**
**50**	**Morokoff, P. J. and LoPiccolo, J. (1986). A comparative evaluation of minimal therapist contacts and 15-session treatment for female orgasmic dysfunction.**	**Journal of Consulting and Clinical Psychology, 54(3), 294.**	**Locke–Wallace Marital Adjustment Test**	**14 couples from the USA (Mean ages for males and females = 33.4 and 30.2 years; Age Ranges = 20–52).**	**Four-session minimal therapist contact (MTC) program for the treatment of lifelong global orgasmic dysfunction in women effectively improved marital happiness. Improving satisfaction with sexual relationships, and, for women in the MTC treatment, happiness in marriage.**			**+ improved marital happiness**	**USA**	**American**	**83**
**51**	**Mujcic, R. and J. Oswald, A. (2016). Evolution of well-being and happiness after increases in consumption of fruit and vegetables.**	**American Journal of Public Health, 106(8), 1504–1510.**	**Household, Income, and Labor Dynamics in Australia Survey (HILDA)**	**12,385 Australian adults (Age Range = 15 to 93 years).**	**Increased fruit and vegetable consumption was predictive of increased happiness, life satisfaction, and well-being. Effects on incident changes in happiness and life satisfaction for people’s changing incomes and personal circumstances. Increased fruit and vegetable consumption was predictive of increased happiness.**			**+ increased fruit and vegetable consumption**	**South Africa**	**South African**	**126**
**52**	**Ngamaba, K. H. (2016). Happiness and life satisfaction in Rwanda.**	**Journal of Psychology in Africa, 26(5), 407–414.**	**Self-report item using 4-point Likert Scale**	**3030 participants from Rwanda (Mean age = 34.2 years, SD = 12.7, Range = 16 to 90 years; 51% Female; 49% Male).**	**Males had greater self-rated happiness than females. State of health, sense of freedom of choice, valuing friends, weekly religious attendance, and national pride positively predicted happiness. The study investigated predictors of happiness and life satisfaction in Rwanda. A fixed effects multilevel regression model was used to predict happiness and life satisfaction from gender, health, socio-economic, and some subjective measures. Happiness is most closely associated with emotions, feelings, or moods (Watson, Clark, and Tellegen, 1988; Gustafsson, Johansson, and Palmer, 2009). Indicators of happiness and life satisfaction may have different salience in developing and developed countries (Howell and Howell, 2008; Fleche, Smith, and Sorsa, 2011; Jorm and Ryan, 2014).**	**+ positive emotions, positive feelings**	**+ freedom of choice**	**+ valuing friends, weekly religious attendance, national pride**	**Rwanda**	**Rwandan**	**26**
**53**	**North, R. J., Holahan, C. J., Moos, R. H., and Cronkite, R. C. (2008). Family support, family income, and happiness: A 10-year perspective.**	**Journal of Family Psychology, 22(3), 475.**	**Self-report happiness item using 5-Point Scale**	**274 participants from the USA (Mean Age = 37, SD = 13.39, Age Range = 18–82 years; 48% Female, 52% Male).**	**Income had a small positive impact on happiness. Family social support, i.e., cohesion, expressiveness, and conflict, showed a positive association with concurrent happiness. The authors used hierarchical linear modeling to investigate the relationship between family income and happiness. Cohesion, expressiveness, and conflict showed a substantial, positive association with concurrent happiness. Income and happiness are most strongly associated at lower levels of income, and higher income categories are associated with ever smaller increments in happiness (Biswas-Diener, 2002).**		**+ income, expressiveness**	**+ family support, social support, cohesion**	**USA**	**American**	**52**
**54**	**Panadero, S., Guillén, A. I., and Vázquez, J. J. (2015). Happiness on the street: Overall happiness among homeless people in Madrid (Spain).**	**American Journal of Orthopsychiatry, 85(4), 324.**	**Overall, Happiness Faces Scale (FS)**	**235 homeless adults from Spain (Mean age = 47.64, SD = 11.94; 17% Female, 83% Male).**	**A positive perceived general health was associated with higher overall happiness, whereas feelings of loneliness and disability/handicap were associated with lower overall happiness. They tested a hypothesized model of overall happiness among homeless people in Spain. The results obtained show that around half of the homeless people in Madrid said that they were happy. Happiness also showed a significant effect on future expectations. The research shows a positive relationship between health and happiness**	**+ general health, health − disability, handicap, positive image,**	**+ future expectations**	**+ homeless people, − feelings of loneliness**	**Spain**	**Spanish**	**54**
**55**	**Park, H. I., Monnot, M. J., Jacob, A. C., and Wagner, S. H. (2011). Moderators of the relationship between person-job fit and subjective well-being among Asian employees.**	**International Journal of Stress Management, 18(1), 67.**	**Happiness Measure (HM)**	**90 Asian employees in US (Mean age = 34.70, SD = 7.35; 73% Female, 27% Male).**	**High core self-evaluation and needs-supplies fit (congruence between employees’ needs and the rewards received for work) significantly predicted greater happiness. Employees with a high person–organization fit displayed greater increases in happiness than employees with low person–organization fit. In Study 1 (N = 90), the interaction between core self-evaluation and needs-supplies (N-S) fit significantly predicted happiness but not depression. Workers with high levels of core self-evaluation were less affected by N-S fit, whereas employees who had low core self-evaluation displayed a reduced level of happiness when there was a lack of N-S fit. Happiness (vs. unhappiness) involves a purely evaluative type of well-being**	**+ high core self-evaluation,**	**+ high needs-supplies fit, high person-organization fit, − low self-evaluation**		**Asia**	**Asian**	**57**
**56**	**Peltzer, K., Pengpid, S., Sodi, T., and Mantilla Toloza, S. C. (2017). Happiness and health behaviors among university students from 24 low-, middle- and high-income countries.**	**Journal of Psychology in Africa, 27(1), 61–68.**	**17,508 participants from 24 countries across Asia, Africa, and the Americas (Mean Age = 20.9, SD = 2.8, Age Range = 16–30 years; 58% Female, 42% Male)**	**17,508 participants from 24 countries across Asia, Africa, and the Americas (Mean Age = 20.9, SD = 2.8, Age Range = 16–30 years; 58% Female, 42% Male).**	**Students from countries of the Caribbean, South America, and Sub-Saharan Africa had greater happiness scores than students from countries in North Africa and Asia. The study examined health behaviors and happiness and associated factors in low. The results indicate that the overall happiness mean score among university students across 24 countries was 13.7 (range 4–20) in middle- and high-income countries. In multivariate linear regression analysis, better subjective socio-economic status, coming from a higher income country, higher social support, higher intrinsic religiosity, higher personal mastery, positive health behaviors, and negative health behaviors) were correlated with happiness. The two positive psychological states for happiness include ‘positive affect or hedonic well-being’ (feelings of happiness and enjoyment) and eudaimonia well-being (human self-actualization) (Ryan and Deci, 2001; Steptoe, O’Donnell, Marmot, and Wardle, 2009a).**	**+ positive health behaviors, − negative health behaviors**	**+ higher personal mastery, positive health behaviors**	**+ Better subjective socio-economic status, coming from a higher income country, higher social support, higher intrinsic religiosity**	**24 countries**	**25 countries**	**26**
**57**	**Pholphirul, P. (2014). Healthier and happier? The urban-rural divide in Thailand.**	**Journal of Human Behavior in the Social Environment, 24(8), 973–985.**	**National Survey**	**2402 participants from Thailand (Age Range = 15–60 years, 70% Female, 30% Male).**	**Income did not affect the level of happiness of those who lived in either urban or rural areas. The study specifically investigates the differences between the health and happiness outcomes of people who live in urban and rural areas in Thailand. Income is found to not affect the level of happiness of those who live in either urban or rural areas. The study of happiness involves various academic fields, from psychology to economics. The results of studies using macro-level data such as employment rate, pollution rate, or level of income of a country, suggest that in the past 50 years, even though the average income and consumption level of the world’s population has been continuously increasing, its happiness level has not increased as indicated by the happiness indicator measured by new Economic Foundation. Macro-level data suggest that happiness derives from living in a good society and an environment that fosters generosity and is free from cheating. Micro-data found that, on average, wealthy people are happier than impoverished people. The current study uses nationally surveyed data obtained from the Happiness Indicator of a study called “The Development and Testing of the New Version of Thai Mental Health Indicator” conducted by the Department of Mental Health, Ministry of Public Health. The comparative results here suggest that rural people’s level of life satisfaction is generally higher than that of urban dwellers. Income is found to have no impact on physical and mental health, pleasure, and happiness levels.**			**+ living in a good society, environment that fosters generosity, environment that is free from cheating**	**Thailand**	**Thai**	**45**
**58**	**Powell, L., Chia, D., McGreevy, P., Podberscek, A. L., Edwards, K. M., Neilly, B., ... and Stamatakis, E. (2018). Expectations for dog ownership: Perceived physical, mental, and psychosocial health consequences among prospective adopters.**	**PLoS ONE, 13(7).**	**Self-report Online Questionnaire**	**3465 participants from Australia (Range = 18 to 65+ years; 85% Female; 15% Male).**	**Dog ownership respondents expected increased happiness. The physical, mental, and psychosocial health benefits and challenges they associated with dog ownership. Among the potential benefits, respondents expected increased walking (89%), happiness (89%), and companionship (61%) and decreased stress (74%) and loneliness (61%).**			**+ dog ownership**	**Australia**	**Australian**	**278**
**60**	**Reyes, J. A. L. (2016). Exploring leisure time activities and sociodemographic indicators of subjective happiness and self-perceived health among Filipinos.**	**Austrian Journal of South East Asian Studies, 9(2), 269–288.**	**International Social Survey Programme 2007**	**1200 participants from the Philippines (Age Range = 18–91 years, 50% Female, 50% Male).**	**Age, leisure activity engagement, and the earnings of others in the neighborhood were negatively associated with happiness; subjective economic status/progress and religious activity attendance were positively associated with subjective happiness. The relationships of subjective happiness and self-perceived health were significantly predicted by subjective socio-economic status, but not by actual family income.**		**+ leisure activities, engagement, subjective economic status, − earning of others in the neighborhood**	**+ age, religious activity**	**Philippines**	**Philippines**	
**61**	**Salim, S., Robinson, M., and Flanders, C. E. (2019). Bisexual women’s experiences of microaggressions and microaffirmations and their relation to mental health.**	**Psychology of Sexual Orientation and Gender Diversity, 6(3), 336.**	**5-day daily diary AND single-item happiness questionnaire**	**85 participants from the USA (Mean Age = 24; Age Range = 18 to 58 years; Identified as cisgender women = 70.6%, bisexual = 82.4%, and white only = 62.3%).**	**Daily subtle negative experiences were related to adverse mental health outcomes, such as depression, suicidality, and decreased happiness. There was a significant relationship between daily microaggressions and depression, suicidality, and happiness within individuals.**	**− adverse mental health outcomes, depression, suicidality**			**USA**	**American**	**46**
**62**	**Senasu, K. and Singhapakdi, A. (2017). Determinants of happiness in Thailand: The moderating role of religiousness.**	**Journal of Human Behavior in the Social Environment, 27(4), 270–290.**	**Telephone Questionnaire**	**1004 respondents from Thailand (Age Range = 25 to 54 years; 50% Female; 50% Male).**	**Family satisfaction, health satisfaction, environmental livability, and religiousness positively affected happiness. The family satisfaction aspect played the most important role in predicting happiness. The results verify the importance of family satisfaction, health satisfaction, environmental livability, and religiousness as important factors of happiness. Happiness can be a cultural issue—for one thing, a positive view of the self is instrumental to a person’s happiness (Biswas-Diener and Diener, 2001; Biswas-Diener et al., 2005).**	**+ health satisfaction,**	**+ positive self-view**	**+ family satisfaction, environmental livability, religiousness**	**Thailand**	**Thai**	**45**
**63**	**Severy, L. J., Waszak, C., Badawi, I., and Kafafi, L. (2003). The psychological well-being of women of Menoufiya, Egypt: Relationships with family planning.**	**American Psychologist, 58(3), 218.**	**Center for Epidemiological Studies Depression Scale (CES-D)**	**795 participants from Egypt (Mean Age = 28.9; 100% Female).**	**Using family planning, having a smaller number of children, and women disagreeing with subservient gender attitudes led to higher happiness. The use of family planning and the number of children born within the preceding 5 years predicted the state of the ratings of happiness.**			**+ family planning, number of children, women disagreeing with subservient gender attitudes**	**Egypt**	**Egyptian**	**142**
**64**	**Shams, K. (2014). Determinants of subjective well-being and poverty in rural Pakistan: a micro-level study.**	**Social Indicators Research, 119(3), 1755–1773.**	**Social Well-Being (SWB) Poverty Measure**	**600 participants from Pakistan.**	**Individuals with unemployment and low health status were found in a lower happiness category. Increasing education income, a higher level of education, and family size were found in the high happiness category. Happiness was found to be U-shaped in age. Socio-demographic factors on happiness. This study suggests a positive impact on well-being and poverty. Subjective well-being (SWB) refers in this context to the overall well-being; i.e., happiness in terms of socio-economic status is measured in this paper.**	**− low health status**	**+ increasing education, increasing income, − unemployment**	**+ family size, age**	**Pakistan**	**Pakistani**	**77**
**65**	**Shams, K. and Kadow, A. (2018). Happiness across the life span: Evidence from urban Pakistan.**	**FWU Journal of Social Sciences, 12(1), 17–30.**	**Social Well-Being Scale (SWB)**	**750 participants from Pakistan (Age Range = 14 and above).**	**Happiness increased with the number of children in urban areas of Pakistan. The results moreover suggest a U-shaped age–happiness pattern. Happiness into various variables, for instance, age, gender, income, employment status, and marital status (Diener et al., 1999).**		**+ income, employment**	**+ increase in the number of children, age, marital status**	**Pakistan**	**Pakistani**	**14**
**66**	**Simons, M., Peeters, S., Janssens, M., Lataster, J., and Jacobs, N. (2018). Does age make a difference? Age as moderator in the association between time perspective and happiness.**	**Journal of Happiness Studies, 19(1), 57–67.**	**Subjective Happiness Scale (SHS)**	**530 participants from Netherlands (Mean Age = 49.15, SD = 14.01, Age Range = 20 to 87 years; 68% Female, 32% Male).**	**Time perspective (the predominantly unconscious process of assigning personal and social experiences to time frames, that help individuals organize and value these events) was associated with happiness. With aging, the negative association between a past-negative time perspective (represents a negative and pessimistic attitude towards the past) and happiness weakened. Regression analysis shows that time perspective is indeed associated with happiness. Research shows that time perspective is associated with different aspects of well-being, including perceived happiness (i.e., Boniwell et al., 2010; Hicks et al., 2012; Zimbardo et al., 1997).**			**− aging, past-negative time perspective**	**Netherlands**	**Netherlander**	**82**
**67**	**Speed, D. and Fowler, K. (2017). Good for all? Hardly! Attending church does not benefit religiously unaffiliated.**	**Journal of Religion and Health, 56(3), 986–1002.**	**Self-report happiness item using 5-Point Scale**	**3620 participants from Canada (Age Range = 45–49 years; 55% Female, 45% Male).**	**Religious attendance and religiosity were significant positive predictors of happiness. Identifying as Christian or a non-affiliate was not associated with greater happiness. In non-affiliated groups, attendance, prayer/meditation, and religiosity were not significant predictors of greater happiness. Research showed the relationships between religious/spiritual variables (attendance, prayer/meditation, and religiosity) and health outcomes (happiness, self-rated health).**			**+ religious attendance, religiosity, attending church**	**Canada**	**Canadian**	**55**
**68**	**Steptoe, A., Leigh, E. S., and Kumari, M. (2011). Positive affect and distressed affect over the day in older people.**	**Psychology and Aging, 26(4), 956.**	**Self-report happiness item using 4-Point Scale**	**4258 participants from the UK (Mean Age = 64.3, SD = 7.4, Age Range = 52–79 years; 55% Female, 45% Male).**	**The associations of loneliness highlight the everyday distress and reduced happiness and excitement experienced by lonely older men and women, and these may contribute to enhanced risks to physical and mental health.**			**+ loneliness**	**UK**	**British**	**46**
**69**	**Sujarwoto, S. and Tampubolon, G. (2015). Decentralisation and citizen happiness: A multilevel analysis of self-rated happiness in Indonesia.**	**Journal of Happiness Studies, 16(2), 455–475.**	**Indonesian Family Life Survey**	**29,024 participants from Indonesia (Mean Age = 37, SD = 17, Age Range = 15–90 years; 52% Female, 48% Male).**	**Fiscal decentralization (improved capacity of districts to deliver public services) significantly increased citizen happiness. Multilevel analysis is used to examine the effect of fiscal and political decentralization on citizen happiness and shows that fiscal decentralization is significantly associated with citizen happiness.**		**+ fiscal decentralization**	**+ political decentralization**	**Indonesia**	**Indonesian**	**82**
**70**	**Surinrut, P., Auamnoy, T., and Sangwatanaroj, S. (2016). Enhanced happiness and stress alleviation upon insight meditation retreat: mindfulness, a part of traditional Buddhist meditation.**	**Mental Health, Religion and Culture, 19(7), 648–659.**	**Thai Happiness Indicators (THI-15)**	**330 participants from Thailand (Mean age = 57.8, SD = 7.52, Age Range = 45–80 years; 84% Female, 16% Male).**	**Meditation interventions (seven-day intensive Vipassana retreat) enhanced happiness. The effect sizes for happiness and perceived stress were 0.379 and −0.428 in the meditation group. The meditation group showed a significant increase in happiness**	**+ meditation**			**Thailand**	**Thai**	**26**
**71**	**Tang, T. L. P., Sutarso, T., Ansari, M. A., Lim, V. K. G., Teo, T. S. H., Arias-Galicia, F., ... and Vlerick, P. (2018). Monetary intelligence and behavioral economics across 32 cultures: Good apples enjoy good quality of life in good barrels.**	**Journal of Business Ethics, 148(4), 893–917.**	**General Social Survey (GSS)**	**6586 participants from 32 cultures across 6 continents (Mean Age = 34.66; 49% Female, 51% Male).**	**Neither GDP nor income were related to happiness. In low GDP (poor) entities, high income was related to escalated happiness. Neither GDP nor income is related to happiness (money makes people happy). The happiness–income paradox exists in developed and developing countries (Easterlin et al. 2010). People with higher incomes have higher levels of happiness compared to others in their society.**		**+ high income in low GDP entities**		**32 countries**	**33 countries**	**176**
**72**	**Tumen, S. and Zeydanli, T. (2015). Is happiness contagious? Separating spillover externalities from the group-level social context.**	**Journal of Happiness Studies, 16(3), 719–744.**	**General Health Questionnaire (GHQ)**	**97,372 participants from the UK (Mean age = 39.59 years, SD = 12.60; 49% Female; 51% Male).**	**Group-level happiness does not have a statistically significant endogenous effect on individual-level happiness. Group-level happiness is neither negative nor positive on the individual level. Individual-level happiness is instead determined by social context (age, education, employer status, and health) in Great Britain. These results suggest that higher group-level happiness does not spill over to the individual level in either a negative or a positive sense. Studies find that “clusters of happiness result from the spread of happiness” [see, e.g., Fowler and Christakis (2008)].**	**+ health**	**+ education, employer status**	**+ social context**	**UK**	**British**	**82**
**73**	**Waldinger, R. J. and Schulz, M. S. (2010). What’s love got to do with it? Social functioning, perceived health, and daily happiness in married octogenarians.**	**Psychology and Aging, 25(2), 422.**	**Self-report happiness item using 7-Point Scale**	**47 older heterosexual couples from the US (Mean age for men = 82.9, SD = 1.7; Age Range 80–88, Mean Age for women = 78.8, SD = 6.16; Age range 62–87).**	**Marital satisfaction protected older adults’ happiness from daily fluctuations in perceived physical health, and social connections promoted happiness in older adults. For both men and women, marital satisfaction buffered day-to-day links between poorer perceived health and a decline in happiness. Physical health and social functioning are widely believed to be important determinants of happiness in old age (Jopp and Rott, 2006)**			**+ marital satisfaction, social connections**	**US**	**American**	**46**
**74**	**Wang, J. H. (2015). Happiness and Social Exclusion of Indigenous Peoples in Taiwan-A Social Sustainability Perspective.**	**PLoS ONE, 10(2).**	**Social Change and Policy of Taiwanese Indigenous Peoples Survey (TIPS)**	**2057 participants from Taiwan (Mean Age = 42.33, SD = 12.26, Age Range = 18–65; 56% Female, 44% Male).**	**Mountain indigenous peoples, females, the elderly, those who were healthier, wealthier, highly educated, possessed western beliefs, who received medical benefits, were without housing problems or financial difficulties were more likely to be happy. Happiness and social inclusion are important indicators of social sustainability, as recommended in the Sustainable Development Goals.**		**+ medical benefits, without housing problems, without financial difficulties**	**+ mountain indigenous people, healthy females, healthy elders, posseting western beliefs,**	**Taiwan**	**Taiwanese**	**278**
**75**	**Yadav, P., Nunia, S., Bansal, A., Sureka, S. K., Jena, R., Ansari, M. S., and Srivastava, A. (2017). Multidimensional assessment of quality of life of children and problems of parents in Indian society after pediatric renal transplant: Beyond the Conventional Thoughts.**	**Pediatric Transplantation, 21(6), e13001.**	**Happiness Questionnaire (HQ)**	**62 patients from India (Mean Age = 13.4, SD = 4.1; 29% Female, 71% Male).**	**Greater happiness reported post-renal transplant. Feelings of happiness and peer group socialization were reported in 81% and 69% of patients, respectively. The improved physical health of children post-transplantation is accompanied by a marked improvement in feelings of happiness.**	**+ post-renal transplant, improved physical health**		**+ peer group socialization**	**India**	**Indian**	**30**
**76**	**Yao, Y. F. and Chen, K. M. (2017). Effects of horticulture therapy on nursing home older adults in southern Taiwan.**	**Quality of Life Research, 26(4), 1007–1014.**	**Chinese Happiness Inventory short version (CHI)**	**85 older adults from Taiwan (Mean Age = 77.41, SD = 8.56; Age Range = 65+ years; 61% Female, 39% Male).**	**Horticulture therapy that included plant cultivation and plant-related material application significantly improved happiness. Horticulture therapy improved activities of daily living, happiness, and interpersonal intimacy of older adults in nursing homes.**			**+ horticulture therapy**	**Taiwan**	**Taiwanese**	**77**

(+) indicates positive influence of the determinants with happiness categories (Health, Hope, Harmony). While (−) indicates a negative influence of the determinant on happiness. SD—‘Standard Deviation’.
